# Transcriptome Analysis of Bovine Rumen Tissue in Three Developmental Stages

**DOI:** 10.3389/fgene.2022.821406

**Published:** 2022-03-03

**Authors:** Yapeng Zhang, Wentao Cai, Qian Li, Yahui Wang, Zezhao Wang, Qi Zhang, Lingyang Xu, Lei Xu, Xin Hu, Bo Zhu, Xue Gao, Yan Chen, Huijiang Gao, Junya Li, Lupei Zhang

**Affiliations:** ^1^ Institute of Animal Science, Chinese Academy of Agricultural Sciences, Beijing, China; ^2^ Institute of Animal Husbandry and Veterinary Research, Anhui Academy of Agricultural Sciences, Hefei, China

**Keywords:** rumen development, carcass traits, beef cattle, transcriptome, GWAS enrichment analysis

## Abstract

Rumen development is a crucial physiological challenge for ruminants. However, the molecular mechanism regulating rumen development has not been clearly elucidated. In this study, we investigated genes involved in rumen development in 13 rumen tissues from three developmental stages (birth, youth, and adult) using RNA sequencing. We identified that 6,048 genes were differentially expressed among three developmental stages. Using weighted correlation network analysis, we found that 12 modules were significantly associated with developmental stages. Functional annotation and protein–protein interaction (PPI) network analysis revealed that *CCNB1*, *CCNB2*, *IGF1*, *IGF2*, *HMGCL*, *BDH1*, *ACAT1*, *HMGCS2*, and *CREBBP* involved in rumen development. Integrated transcriptome with GWAS information of carcass weight (CW), stomach weight (SW), marbling score (MS), backfat thickness (BFT), ribeye area (REA), and lean meat weight (LMW), we found that upregulated DEGs (fold change 0∼1) in birth–youth comparison were significantly enriched with GWAS signals of MS, downregulated DEGs (fold change >3) were significantly enriched with GWAS signals of SW, and fold change 0∼1 up/downregulated DEGs in birth–adult comparison were significantly enriched with GWAS signals of CW, LMW, REA, and BFT. Furthermore, we found that GWAS signals for CW, LMW, and REA were enriched in turquoise module, and GWAS signals for CW was enriched in lightgreen module. Our study provides novel insights into the molecular mechanism underlying rumen development in cattle and highlights an integrative analysis for illustrating the genetic architecture of beef complex traits.

## Introduction

Rumen development is an important physiological challenge for young ruminants (Lin et al., 2019). At birth, rumen is incompletely developed without high ketogenic capacity (Warner et al., 1956; Lin et al., 2020). With the rumen development and microbial colonization, calves began to transit from a milk-based diet to a grain and forage-based diet (Jami et al., 2014; Shabat et al., 2016). The development of the rumen during this transition involves three simultaneous processes (Li et al., 2019). First is the physical development of rumen including growth in rumen volume and papilla (Reynolds et al., 2004). Second, microbial communities were established and colonized (Fouts et al., 2012; Rey et al., 2014), which is highly correlated with important carcass traits, such as marbling score (MS), adjusted 12th rib fat thickness, longissimus lipid content, and carcass yield grade (Krause et al., 2020). Meanwhile, there is a functional development of fermentation capacity and enzyme activity in the rumen lumen and epimural layers (Rey et al., 2012). This whole process is not instantaneous, and the ability to ferment solid diet acquired at least 1 week after weaning (Quigley et al., 1985). However, little is known about transcriptome characteristics during these morphological changes.

Previous studies focused on the effects of nutrient, diet composition, or feeding strategy on rumen development. Encouraging calves to consume dry feedstuffs at an early age will accelerate rumen development, allowing more efficient body growth and development at maturity (Coverdale et al., 2004; Norouzian et al., 2011). Commonly rumen microorganisms proliferate and produce energy in the form of volatile fatty acids (VFAs), primarily propionate and butyrate (Reddy et al., 2017). VFAs, particularly butyrate, can stimulate papilla growth, accelerate rumen motility, and muscle growth (Tamate et al., 1962; Kristensen et al., 2007). With the development of high-throughput RNA sequencing (RNA-seq), it is allowed to investigate gene expression of certain tissue as integrity. In beef cattle, RNA-seq has been utilized to investigate the transcriptome of liver, longissimus dorsi muscle, and adipose tissues (Cesar et al., 2015; Huang et al., 2017; Kong et al., 2020). Of these, the developmental transcriptome of ruminal tissue in beef cattle was limitedly reported. Identifying genes expression during ruminal tissue development represents an important step toward understanding the biological processes of rumen growth.

Here, we performed RNA-seq to examine transcriptome profiles and identify candidate genes from rumen tissue of three developing stages. We believed that these results could help us understand the molecular mechanism of rumen development and further illuminate the relation between transcriptome of rumen development and beef complex traits.

## Materials and Methods

### Phenotypes and Rumen Tissue Collection

A total of 13 Simmental half-sib individuals from three periods (birth, youth, and adult) were used in this study, including five calves at birth, five youth individuals (6 months old), and three adult cattle (18 months old). These cattle were raised under the same feeding strategies and conditions in Shayang Hanjiang cattle Co., Ltd. (Shayang County, Jingmen City, Hubei Province). After slaughtering, a 2-cm^2^ piece of rumen tissue was isolated immediately, rinsed with sterilized PBS buffer (pH = 6.8) and placed in a 2-ml tube. All samples were immediately frozen with liquid nitrogen for total RNA extraction.

### Nucleic Acid Extraction, Sequencing, and Genotyping

Total RNA was extracted from rumen tissue using TRIzol reagent (Invitrogen, Life Technologies) according to the protocol of instruction. Total RNA samples were assessed for RNA integrity using the RNA Nano 6000 Assay Kit of the Bioanalyzer 2100 System (Agilent Technologices, CA, USA). RNA concentration was assayed by Qubit, and purity was assayed by Nanophotometer Spectrophotometer (Theermo Fisher Scientific, MA, USA). RNA-seq library construction was performed only for samples with RNA integrity greater than 7. The cDNA library was constructed using the Illumina TruSeqTM RNA Kit (Illumina, San Diego, CA, USA) according to the manufacturer’s instructions. RNA-seq was performed on Illumina NovaSeq 6000 platform using a pairing end strategy [read length 150 base pairs (bp)].

### Sequencing Data Analysis

The clean data were mapped to the reference genome (*Bos taurus* ARS-UCD1.2) by the HISAT2 (v2.2.1) (Kim et al., 2015). Effective reads aligned with gene regions were statistically calculated on the basis of genomic location information specified by bovine reference genome annotations (http://ftp.ensembl.org/pub/release-103/gtf/bos_taurus/). SAMtools (v1.9) (Li et al., 2009) was used to sort the BAM alignment files that were generated from HISAT2 by name. FeatureCounts (v2.0.1) (Liao et al., 2014) and Stringtie (v2.1.1) were used to estimate read counts and normalize reads as transcripts per kilobase of exon model per million mapped reads (TPM) for each sample (Pertea et al., 2015).

### Differentially Expressed Genes Analysis

To investigate differentially expressed genes (DEGs) among three periods (birth vs. youth, birth vs. adult, and youth vs. adult), using DESeq2 (v1.30.1) to normalize the gene count data and calculate differential expression (Love et al., 2014). Genes with a Benjamini–Hochberg adjusted *p*-value < 0.05 were designated as differentially expressed.

### Co-Expression Network Analysis

The DEGs from three comparisons were put together and removed redundant duplicate genes and using R package WGCNA to construct co-expression network (Langfelder and Horvath 2008). Co-expressed gene modules were detected by dynamic tree cutting method (Langfelder et al., 2008). Then, modules with highly correlated GS and MM values (*p*-value ≤ 0.05) and module–trait relationships with a correlation coefficient >0.5 were identified as stage-specific modules (Zhang et al., 2017).

### Function Enrichment and PPI Analysis

To understand the function of genes in each stage-specific module, DAVID (https://david.ncifcrf.gov/) (Huang et al., 2007) and KOBAS (http://kobas.cbi.pku.edu.cn/kobas3/) (Xie et al., 2011) were used to GO enrichment analysis and KEGG pathway analysis, respectively. GO terms and pathways with a *p*-value less than 0.05 were defined as significantly enriched. Genes in each stage-specific module were calculated separately by STRING (https://string-db.org/) (Szklarczyk et al., 2015), obtained the protein–protein interaction (PPI) network, and imported into Cytoscape (v3.8.2). Cytoscape depends on the “Analyze Network” function to get the degree of a node, which means how many edges connect to it (Shannon et al., 2003).

### The Enrichment Analysis of GWAS Signals

Our resource population included 1,478 Simmental beef cattle, which were born between 2008 and 2020 from in Wulagai, Inner Mongolia. All individuals were slaughtered at an average age of 20 months; four carcass traits [carcass weight (CW), lean meat weight (LMW), backfat thickness (BFT), and ribeye are (REA)], MS, and stomach weight (SW) traits were measured. Of these, SW is the total weight of the rumen, reticulum, and abomasum. All phenotypes were adjusted for the environmental fixed effects, including farm, year and sex, pre-fattening weight, and fattening days that were regarded as covariates. The DNA for each animal was isolated from blood samples and genotyped with Illumina BovineHD770kBeadchip (Illumina, San Diego, CA, USA). Before the statistical analysis, SNPs were filtered using PLINK (v1.90) (Purcell et al., 2007). SNPs were removed under strict criteria, and the standards are as follows: minor allele frequency (<0.01), missing genotypes (>0.05), and Hardy–Weinberg equilibrium (*p* < 10^−6^). Consequently, 1,432 individuals and 673,524 SNPs were remained (Xu et al., 2020). The mixed linear model was used for GWAS analysis by the GCTA (v1.93.0) (Yang et al., 2011; Yang et al., 2014) software, and the formula is as follows:

$$Y_{\mathrm{ij}}=b_{j}x_{\mathrm{ij}}+g_{\mathrm{ij}}+e_{\mathrm{ij}}$$

where *y_ij_* is the pre-adjusted phenotypic value of the ith individual with the jth SNP; *b_j_* is the allele substitution effect of SNP j; *x_ij_* is the jth SNP genotype of individual i and *x_ij_* is coded as 0, 1, and 2 for genotypes BB, Bb, and bb; *g_ij_* is the polygenetic effect of the ijth individual, *g_ij_* ∼N (0, σ_a_^2^G), with σ_a_^2^ being the additive genetic variance and G is the additive genetic relationship matrix constructed using all SNPs. *e_ij_* is the residual effect, *e_ij_* *∼N* (0, σ_e_^2^I), with σ_e_^2^ being the residual variance and I is the identity matrix.

In previous reported, rumen microbiota may affect the MS, adjusted 12th rib fat thickness, and other traits (Reddy et al., 2017; Kim et al., 2020), and its fermentation product VFAs can promote muscle growth (Kristensen et al., 2007). To investigate whether DEGs and gene co-expression modules were enriched with GWAS signals of carcass and other traits, we applied a sum-based method for GWAS signals enrichment analyses (sumGSE, https://github.com/WentaoCai/GWAS_enrichment) (Cai et al., 2021), which used signals of all markers within a pre-defined list of DEGs and then calculated the following summary statistics for the DEGs:

$${\text{T}}_{\text{sum}}=\overset{m_{g}}{\underset{i=1}{\sum }}\beta^{2}$$

where T_sum_ was the summary statistics for a tested gene group. m_g_ is the number of SNPs, which is within the DEGs or 5-kb up- and downstream of DEGs, and β was acquired in GWAS statistics, meaning the value of marker effect. We randomly shifted the observed SNPs set to the new positions and calculated their T_sum_ summary statistics. The permutation was repeated 1,0000 times. When formula observed the proportion less than randomly sampled summary statistics, one-tailed tests was applied to calculate the empirical *p*-value.

## Results

### Transcriptome Profiling of Rumen Tissue

**FIGURE 1:**
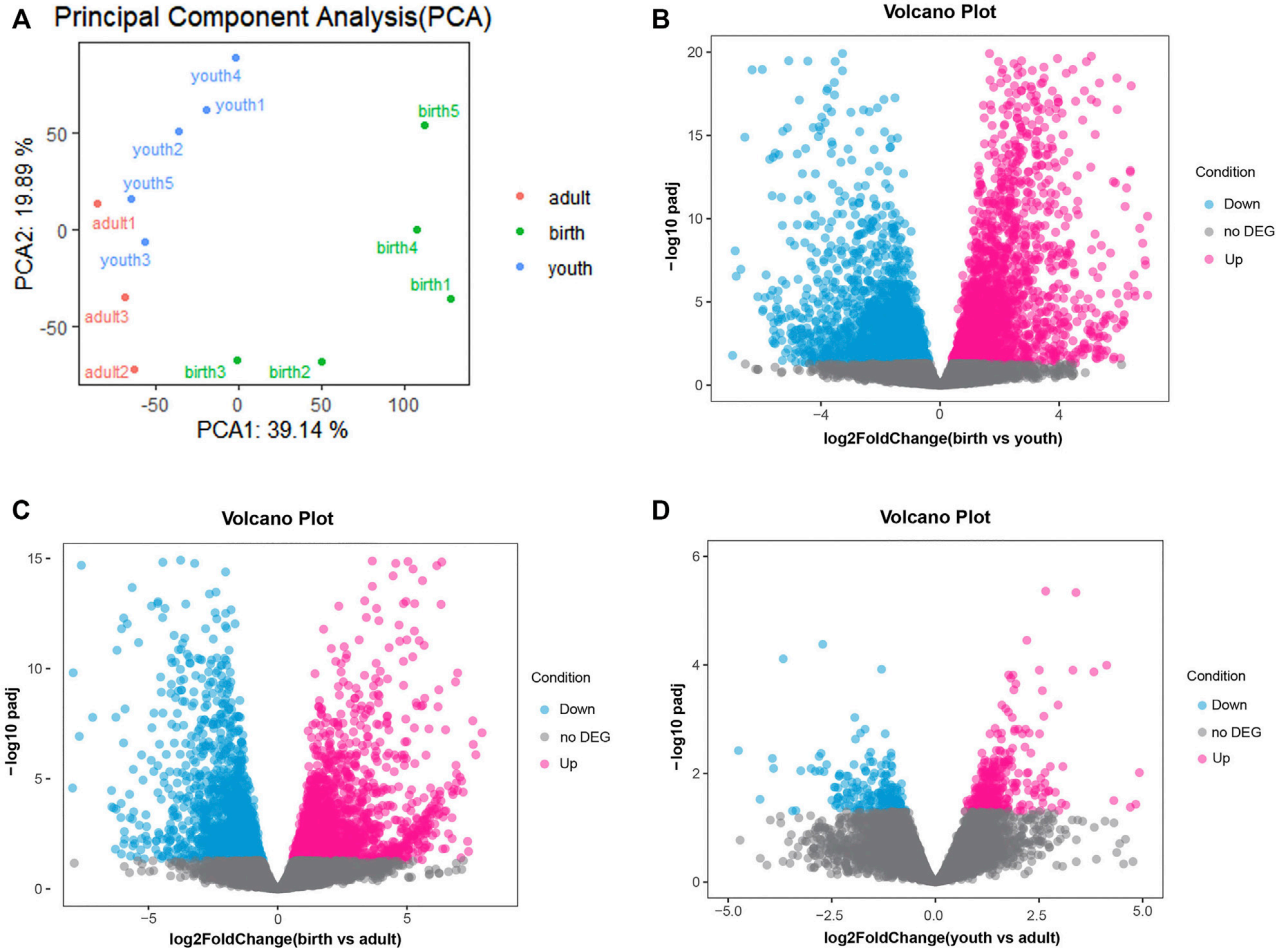
**(A)** PCA of the identified genes, the red, green and blue dots represent samples of adult, birth and youth periods. **(B)** Volcano plot of differential genes, volcano plot for DEGs in rumen tissue comparing birth period and youth period. **(C)** Volcano plot for DEGs in rumen tissue comparing birth period and adult period. **(D)** Volcano plot for DEGs in rumen tissue comparing youth period and adult period, red and blue dots represent up/down-regulated DEGs, respectively. The gray dots represent not DEGs.

A total of 310.8 million raw reads from 13 rumen tissues were generated by RNA-seq. After quality control, we obtained an average of 23.1 million clean reads ranged from 19.1 to 29.0 million reads. The mapping rate was about 96.60% (ranging from 95.41% to 97.51%) after aligning clean reads to the cattle reference genome (ARS-UCD1.2) in Supplementary Table S1. These findings indicated good data quality that were suitable for subsequent analysis. We observed an average of 12,124 genes (ranging from 11,660 to 13,087) that were expressed (TPM > 0 in at least seven samples) across 13 samples. The gene expression of three periods is shown in Supplementary Figure S1, and the samples between youth and adult were highly correlated, while we found obvious differences between birth and youth/adult stage (Supplementary Figure S2). Despite differences in sample characteristics, samples from the same group were clustered together on the basis of their gene expression profiles (Figure 1A).

### Top Genes Expressed in Three Periods

The top 20 expressed genes in the rumen tissue at the birth, youth, and adult stages are shown in Table 1 and Supplementary Table S2. The highest expressed gene in rumen of birth stage was *IRS4*, *GINM1*, *PAQR5*, *S100A14*, *MDP1*, *HOXC4*, *TAF7L*, *RS1*, *FDPS*, *NTS*, *JUP*, *PHB*, *UBE2G1*, *SMS*, *HBS1L*, *CGAS*, *TPM3*, *TUT4*, *GPR153*, and *GLOD4*, whereas *COX1*, *PJA1*, *S100A5*, *S100A12*, *ILF2*, *COX3*, *EFNB1*, *PCYT1B*, *USP11*, *CYTB*, *ND6*, *OPHN1*, *EDA2R*, *SPAG1*, *NEO1*, *ATP6*, *LCTL*, *COX2*, *RPLP1*, *JRKL* and *KRTAP11-1*, *COX1*, *DDX3X*, *S100A12*, *SLC39A1*, *SNORA75*, *COX2*, *COX3*, *ND3*, *ATP6*, *MID1IP1*, *ATP6AP2*, *PIP4K2C*, *RPLP1*, *ATP8*, *CXHXorf38*, *NPR1*, *NOX5*, *DES*, and *CYTB* were highest expressed gene in youth and adult stage, respectively. We found that seven top expressed genes were same between youth and adult, including *COX1*, *S100A12*, *COX3*, *CYTB*, *ATP6*, *COX2*, and *RPLP1*, whereas the top 20 expressed genes of rumen in birth stage were totally different with the other stages, which indicated that the gene expressed pattern of rumen was distinct at birth stage.

**TABLE 1:** Top 20 expressed genes in the rumen tissues at three periods.

Period	Top 20 expressed genes
Birth	IRS4, GINM1, PAQR5, S100A14, MDP1, HOXC4, TAF7L, RS1, FDPS, NTS, JUP, PHB, UBE2G1, SMS, HBS1L, CGAS, TPM3, TUT4, GPR153, GLOD4
Youth	COX1, PJA1, S100A5, S100A12, ILF2, COX3, EFNB1, PCYT1B, USP11, CYTB, ND6, OPHN1, EDA2R, SPAG1, NEO1, ATP6, LCTL, COX2, RPLP1, JRKL
Adult	KRTAP11-1, COX1, DDX3X, S100A12, SLC39A1, SNORA75, COX2, COX3, ND3, ATP6, MID1IP1, ATP6AP2, PIP4K2C, RPLP1, ATP8, CXHXorf38, NPR1, NOX5, DES, CYTB

### Differentially Expressed Genes Across Three Periods

Next, differences in gene expression of rumen tissues between different periods were investigated. In the birth vs. youth comparison, 4,905 DEGs were identified, including 2,486 upregulated genes and 2,419 downregulated genes (Figure 1B and Supplementary Table S3). A total of 3,877 DEGs were identified in birth vs. adult comparison, including 1,991 upregulated genes and 1,886 downregulated genes (Figure 1C and Supplementary Table S4). However, there were fewer DEGs identified between youth and adult, and only 521 DEGs were identified, including 314 upregulated genes and 207 downregulated genes (Figure 1D and Supplementary Table S5). The hierarchical clustering heatmap of all DEGs is shown in Supplementary Figure S3.

### Co-Expression Network Construction and Module Detection

To better understand the function of DEGs, we used WGCNA to explore the relationship between DEGs. DEGs with a low expression level (TPM < 0.05) in more than one sample in the same group were removed, obtaining 6048 DEGs for co-expression analysis (Supplementary Table S6). A scale-free network was constructed using blockwise module function, and 6048 genes were divided into 14 modules, the number genes in each module, ranging from 116 in dark red module to 1,421 in turquoise module (Supplementary Figure S4 and Supplementary Table S7).

### Period-specific Module Identification

**FIGURE 2:**
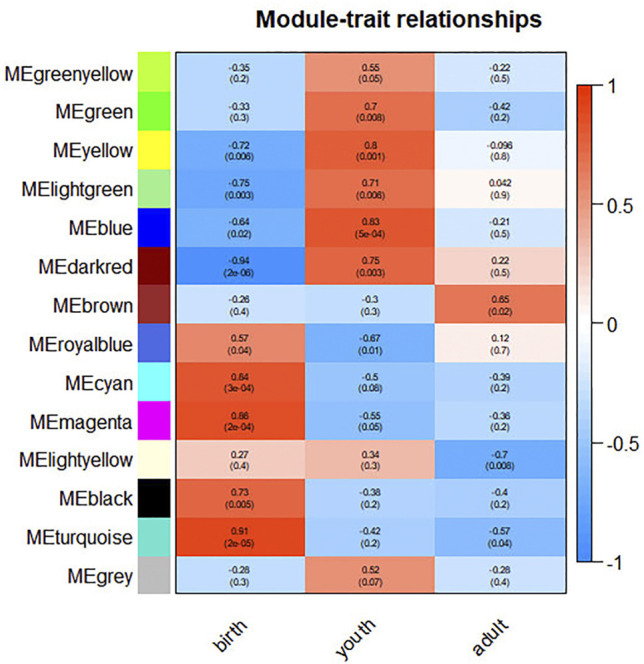
Correlation between modules and differentiation period. The color, ranging from blue through white to red, indicates negative to positive correlation.

GS and MM of all genes in the module were calculated to investigate the period-specific modules during rumen development (Supplementary Figure S5). GS was defined as the correlation between the gene and the developing period. MM was defined as the correlation between module eigengene and the gene expression profile. A strong correlation between GS and MM (*p* ≤ 0.05) shows that genes highly associated with a trait are often the most important elements of the modules associated with that trait. Furthermore, we use ME to represent the gene expression level in each module. The correlations between ME and the period of differentiation were analyzed (Figure 2). Ultimately, we identified 12 period-specific modules (average module–trait relationship > 0.5 and *p* ≤ 0.05), among which the modules royal blue, cyan, magenta, black, and turquoise were positively correlated with birth period; the green yellow, green, yellow, lightgreen, blue, and dark red modules were positively correlated with youth period; and the brown ones were positively correlated with adult period. In contrast, the modules yellow, lightgreen, blue, and dark red were negatively correlated with birth period, and the modules royal blue and turquoise were negatively correlated with youth and adult period, repectively.

### Visualization Potential Genes and Function Enrichment Analysis

**FIGURE 3:**
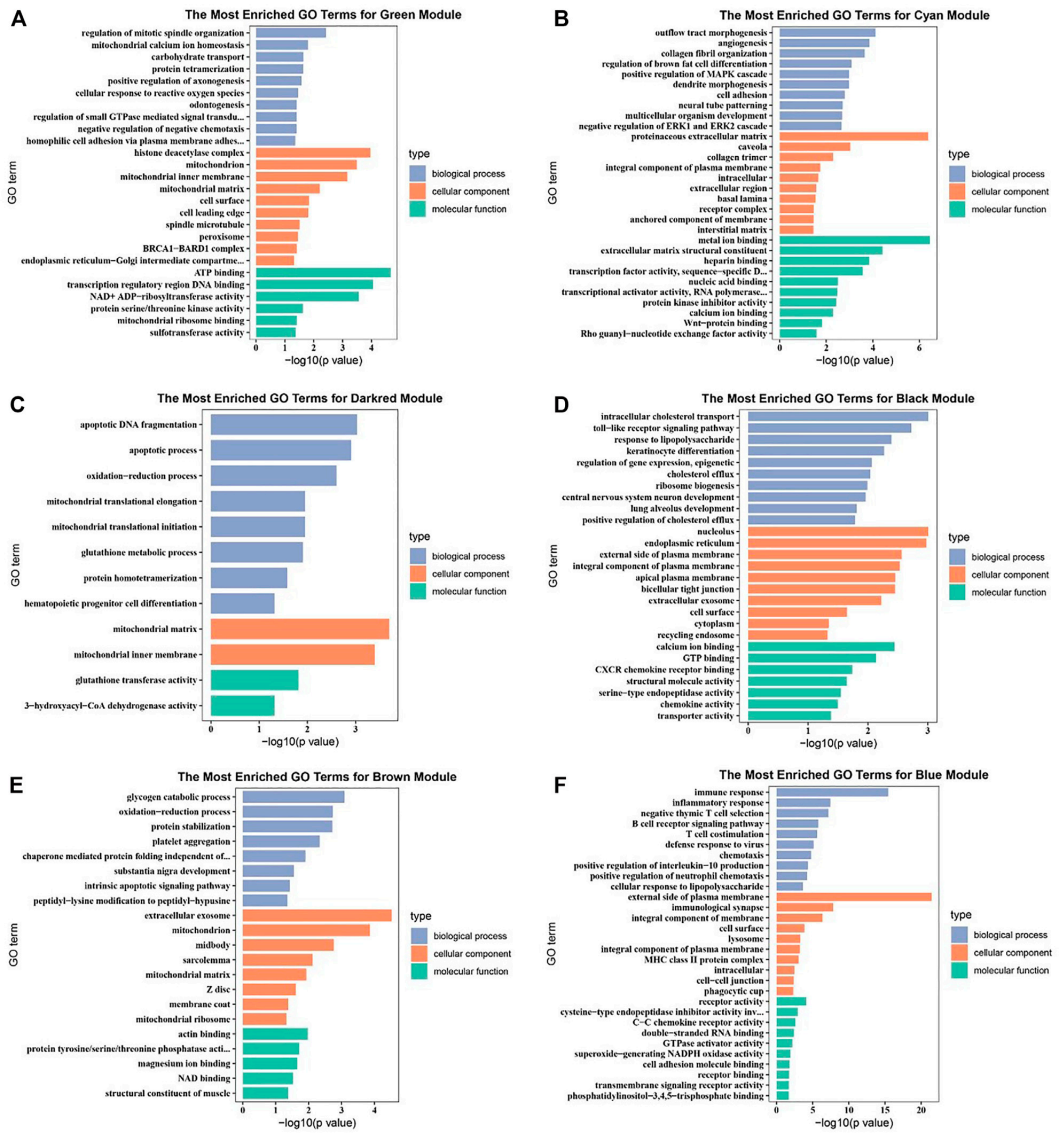
Gene ontology (GO) enrichment analysis of related differentially expressed genes (DEGs). **(A)** GO enrichment analysis of DEGs in green module. **(B)** GO enrichment analysis of DEGs in cyan module. **(C)** GO enrichment analysis of DEGs in darkred module. **(D)** GO enrichment analysis of DEGs in black module. **(E)** GO enrichment analysis of DEGs in brown module. **(F)** GO enrichment analysis of DEGs in blue module.

**FIGURE 4:**
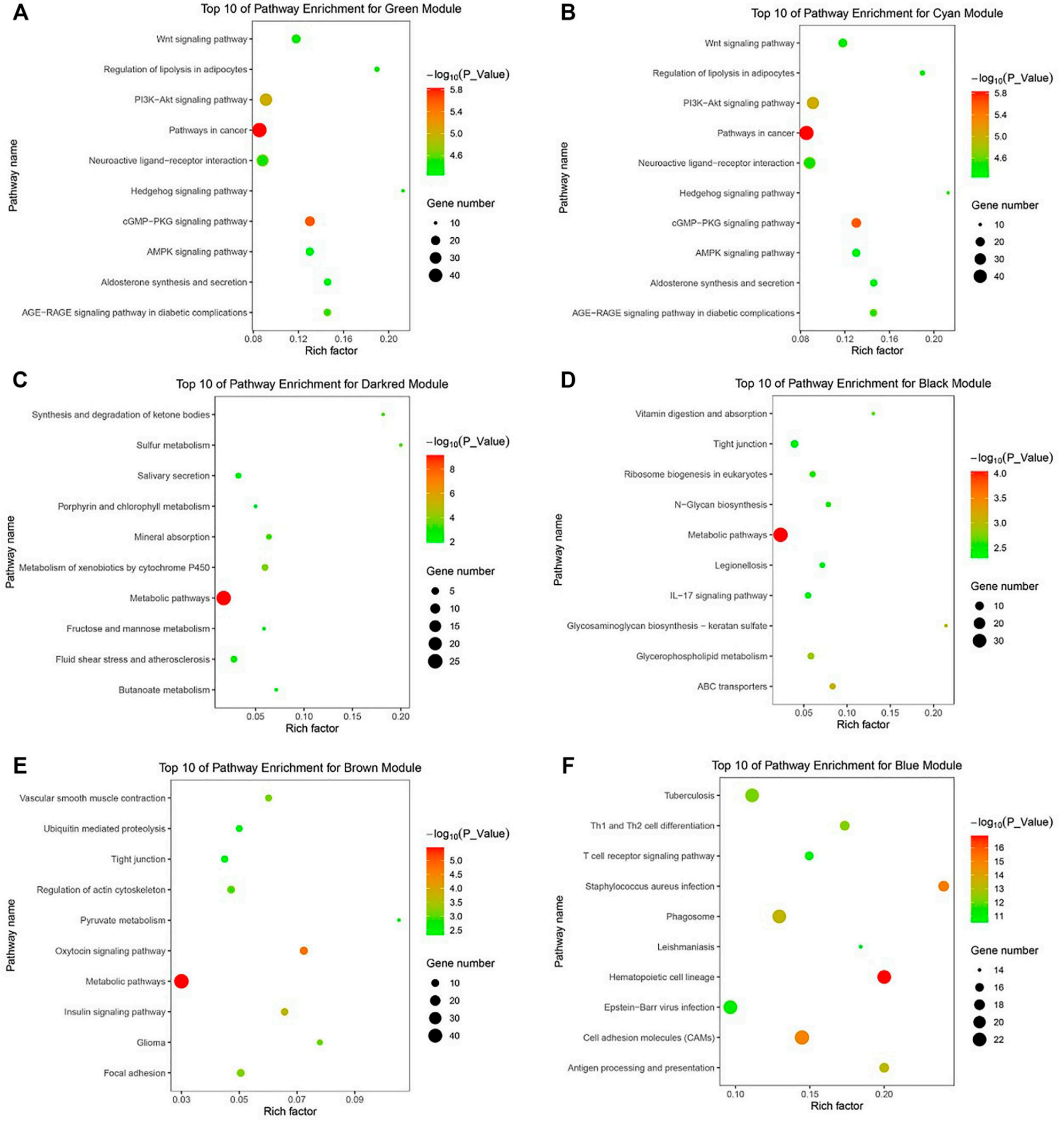
KEGG pathway analysis of related DEGs. **(A)** The top 10 of pathway enrichment for green module. **(B)** The top 10 of pathway enrichment for cyan module. **(C)** The top 10 of pathway enrichment for darkred module. **(D)** The top 10 of pathway enrichment for black module. **(E)** The top 10 of pathway enrichment for brown module. **(F)** The top 10 of pathway enrichment for blue module.

Potential genes were obtained through PPI network and then visualized by Cytoscape (Supplementary Figure S6). The significantly enriched GO terms and the top 10 pathways of each module are shown in Figures 3 and 4 and Supplementary Figures S7 and S8. Detailed information of the GO terms and pathways is shown in Supplementary Tables S8, S9. For rumen development, the important pathways identified were butanoate metabolism, synthesis and degradation of ketone bodies, cell cycle, Wnt signaling pathway, MAPK signaling pathway, PI3K-Akt signaling pathway, and TGF-beta signaling pathway. Multiple significant GO terms are related to cell division, mitochondrial matrix, mitochondrial inner membrane, histone acetyltransferase complex, histone acetyltransferase activity, regulation of multicellular organism growth, positive regulation of phosphatidylinositol 3-kinase signaling, and positive regulation of MAPK cascade. Combining the expression level of DEGs, GO, and pathway results allows us to suggest *CCNB1*, *CCNB2*, *IGF1*, *IGF2*, *HMGCL*, *BDH1*, *ACAT1*, *HMGCS2*, and *CREBBP* as the promising candidate genes for rumen development. Potential genes related to rumen development and their enriched pathways and GO terms are shown in Tables 2 and 3.

**TABLE 2:** Description of potential DEGs that associated with rumen development from different module.

Gene name	Gene full name	Module
IGF1	Insulin-like growth factor I	Cyan
IGF2	Insulin-like growth factor II	Cyan
BDH1	3-Hydroxybutyrate dehydrogenase 1	Darkred
HMGCL	Hydroxymethyl-3-methylglutaryl-CoA lyase	Darkred
CCNB1	Cyclin B1	Lightgreen
CCNB2	Cyclin B2	Lightgreen
ACAT1	Acetyl-CoA acyltransferase 1	Lightgreen
HMGCS2	3-Hydroxy-3-methylglutaryl-CoA synthase2	Lightgreen
CREBBP	CREB Binding Protein	Turquoise

**TABLE 3:** Various GO terms and pathways related to rumen development shown from different modules.

GO term/pathway	ID	Gene	Module
Cell division	GO: 0051301	CCNB1, CCNB2	Lightgreen
Mitochondrial matrix	GO: 0005759	BDH1	Darkred
Mitochondrial inner membrane	GO: 0005743	HMGCL, HMGCS2, ACAT1	Darkred, lightgreen
Histone acetyltransferase complex	GO:0000123	CREBBP	Turquoise
Histone acetyltransferase activity	GO:0004402	CREBBP	Turquoise
Positive regulation of MAPK cascade	GO: 0043410	IGF2	Cyan
Regulation of multicellular organism growth	GO: 0040014	IGF1	Cyan
Positive regulation of phosphatidylinositol 3-kinase signaling	GO: 0014068	IGF1	Cyan
Cell cycle	bta04110	CCNB1, CCNB2, CREBBP	Lightgreen
Butanoate metabolism	bta00650	HMGCL, BDH1, ACAT1, HMGCS2	Darkred, lightgreen
Wnt signaling pathway	bta04310	CREBBP	Turquoise
MAPK signaling pathway	bta04010	IGF1, IGF2	Cyan
Synthesis and degradation of ketone bodies	bta00072	HMGCL, BDH1, ACAT1, HMGCS2	Darkred, lightgreen
PI3K-Akt signaling pathway	bta04151	IGF1, IGF2	Cyan
TGF-beta signaling pathway	bta04350	CREBBP	Turquoise

### Different Group and Module Genes in the Enrichment of Association Signals

**FIGURE 5:**
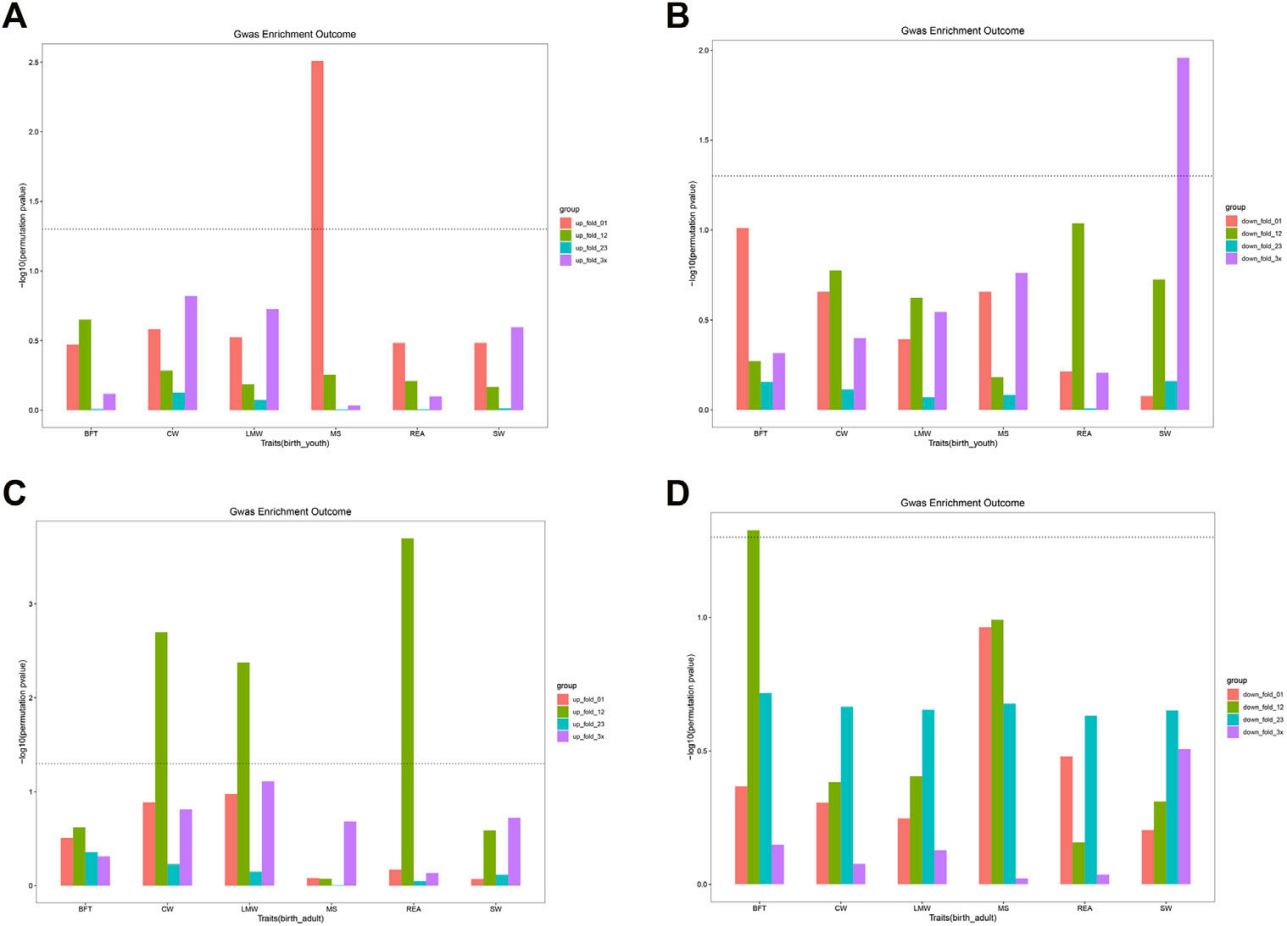
The GWAS enrichment for DEGs in different groups. **(A)** The enrichment for DEGs of four groups in birth vs youth. **(B)** The enrichment for DEGs of four groups in birth vs youth. **(C)** The enrichment for DEGs of four groups in birth vs adult. **(D)** The enrichment for DEGs of four groups in birth vs adult. The line means P is equal to 0.05.

To investigate whether DEGs and gene co-expression modules were enriched with GWAS signals of carcass and beef quality traits, we applied enrichment analysis for all DEGs and modules across six traits (Tables 4, 5). As shown in Supplementary Figure S9A, DEGs were significantly enriched with GWAS signals of CW and LMW. In birth–youth comparison, fold change 0∼1 (slightly) upregulated DEGs were significantly enriched with GWAS signals of MS (Figure 5A), and fold change >3 (dramatically) downregulated DEGs were significantly enriched with GWAS signals of SW (Figure 5B). In birth–adult comparison, fold change 1∼2 up/downregulated DEGs were significantly enriched with GWAS signals of CW, LMW, REA, and BFT (Figures 5C,D). In youth–adult comparison, DEGs were not significantly enriched with GWAS signals. Furthermore, as shown in Supplementary Figure S9B, DEGs of turquoise module were significantly enriched with GWAS signals of CW, LMW, and REA; DEGs of lightgreen module were significantly enriched with GWAS signals of CW; and DEGs of other modules were not significantly enriched with GWAS signals (Supplementary Figures S9C,D). Of these, *GOLGB1*, *ACVR2A*, and *TWIST2* belong to turquoise module and slightly upregulated DEGs, which indicated that *GOLGB1* and *ACVR2A* might affect CW traits and *TWIST2* might affect REA trait. Furthermore, *IGF2* belongs to dramatically downregulated DEGs and might affect SW trait.

**TABLE 4:** The GWAS enrichment for DEGs in different groups and modules.

Trait	Group/module	*p*-value
A		
CW	birth_adult	0.0039
LMW	birth_adult	0.0066
B		
MS	up_fold_01	0.0031
SW	down_fold_314	0.0110
C		
LMW	up_fold_12	0.0042
CW	up_fold_12	0.0020
REA	up_fold_12	0.0002
BFT	down_fold_12	0.0471
D		
CW	Turquoise	0.0232
REA	Turquoise	0.0322
LMW	Turquoise	0.0296
CW	Lightgreen	0.0231

A. The three periods are compared in pairs. B. In birth_youth comparison, DEGs were divided into upregulated and downregulated genes, which were further divided into up/down_fold_01, up/down_fold_12, up/down_fold_23, and up/down_fold_3x according to fold change. C. In birth_adult comparison, the grouping principle is the same as that of B. D. DEGs were segmented by modules.

**TABLE 5:** Statistical description of six traits in Chinese Simmental beef cattle.

Traits	*N*	Mean (SD)	Min	Max
CW	1,471	284.40 ± 54.51	162.60	448.12
MS	1,436	5.19 ± 0.82	3.00	6.00
SW	1,059	8.45 ± 2.01	4.72	15.71
BFT	839	3.11 ± 2.68	0.05	12.50
REA	1,331	87.28 ± 14.39	51.00	133.00
LMW	1,456	240.26 ± 46.53	126.00	383.00

CW, carcass weight; MS, marbling score; SW, stomach weight; BFT, backfat thickness; REA, ribeye area; LMW, lean meat weight.

## Discussion

In this study, we obtained a comprehensive landscape of transcriptome profiles across 13 rumen tissue samples during three different stages of growth. Importantly, we identified candidate genes and networks related to rumen development. We obtained top 20 genes expressed in the rumen tissue during three periods. Of these, *COX1*, *COX2*, *COX3*, *CYTB*, *ATP6*, *RPLP1*, and *S100A12* have occurred in both youth and adult period. *COX1*, *COX2*, and *COX3* are three subunits of cytochrome c oxidase that may play an important role in the production of vast majority of ATP molecules in mammalian cells (Čunátová et al., 2020). *CYTB* is involved in electron transport in the mitochondrial respiratory chain (Seddigh and Darabi 2018). *ATP6* has transmembrane transport activity of hydrogen ions (Li et al., 2016). *RPLP1* plays an important role in the elongation step of protein synthesis (Du et al., 2007). *S100A12* is involved in cell cycle progression and differentiation (Fritz et al., 2010). These genes and its functions indicated that two periods are associated with energy and cell proliferation. Furthermore, the top 20 expression genes of birth period had no common genes with other periods, but *S100A14* in birth period and *S100A5* and *S100A12* in youth or adult periods belong to the same gene family that functions in cell cycle progression and differentiation (Liu et al., 2008).

Rumen development is regulated by a various of factors. Previous studies indicated the genes of *FABP7*, *ILK*, *PDGFɑ*, *HMGCS2*, *FABP3*, and *AKR1C1* involved in rumen development (Kato et al., 2016; Malmuthuge et al., 2019). Moreover, CREBBP, TTF2, TGFB1, and PPARɑ are capable of contributing to the development of rumen epithelium (Baldwin et al., 2012; Connor et al., 2013; Connor et al., 2014). In our study, we identified that *CCNB1*, *CCNB2*, *IGF1*, *IGF2*, *HMGCL*, *BDH1*, *ACAT1*, *HMGCS2*, and *CREBBP* are the potential genes involved in rumen development, some of which were reported previously. HMGCS2 plays an important role in ketogenesis in the rumen epithelium of sheep during development (Lane et al., 2002). Furthermore, HMGCS2 was identified as a downstream target of PPAR*α*. Increased production of VFA induced by intake of solid feed during weaning might promote ketogenesis in rumen epithelial cells. In this progress, PPAR*α* promoted papillary development by activating *HMGCS2* (Connor et al., 2013). *ACAT1*, *BDH1*, *HMGCL*, and *HMGCS2* were found involved in the pathways of synthesis and degradation of ketone bodies and butanoate metabolism, which were found as features of rumen wall development (Sun et al., 2021). Microbial fermentation of dietary carbohydrates in the rumen produces large amount of short-chain fatty acids (SCFAs) (Poulsen et al., 2012), such as butyrate, which are known to affect rumen development and stimulate rumen epithelial cells proliferation *in vivo* (Sakata and Tamate 1978). SCFA transport process involves many regulatory factors including insulin-like growth factor (*IGF*), sodium hydrogen exchangers (*NHE*), monocarboxylate transporters (*MCTs*), and epidermal growth factor (*EGF*), which are also involved in the regulation of rumen epithelial cell proliferation (Baldwin 1999; Yang et al., 2012; Benesch et al., 2014; Nishihara et al., 2020). Butyrate is mainly absorbed and metabolized in the rumen epithelium (Sehested et al., 1999), producing ketone body under the action of ACAT1, BDH1, HMGCS2, and HMGCL (Kostiuk et al., 2010). Of these, HMGCS2 is a rate-limiting mitochondrial enzyme in the ketogenic pathway (Lane et al., 2002) and catalyzes synthesis of 3-hydroxy-3-methylglutaryl-CoA (HMG-CoA), the central metabolite of rumen epithelial cells (Xiang et al., 2016).

Cell proliferation is an important part of rumen development and is affected by various aspects (Naeem et al., 2012). In our study, *CCNB1* and *CCNB2* were enriched in the cell division, cell cycle pathway. Previous study indicated that the *CCNB1* expression promoted ruminal cell cycle progression in goat (Gui and Shen 2016). *CCNB2* was also found playing important roles in the acceleration of cell cycle and rumen development (Cui et al., 2018; Sun et al., 2021). CREBBP functions as a transcriptional coactivator of RNA polymerase II–mediated transcription and plays an important role in cell growth (Kalkhoven 2004; Baldwin et al., 2012). Furthermore, butyrate has been shown to affect the rumen by regulating the transcription factor CREBBP (Baldwin et al., 2012). *CREBBP* was enriched in the cell cycle, TGF-beta signaling pathway, and Wnt signaling pathway, part of which were highly associated with cell proliferation, apoptosis, and differentiation (Huang and Huang 2005; Kabiri et al., 2018; Guo et al., 2021). Kim et al. (2013) reported that the activity of IGF-I or IGF-II can regulate epithelial cell proliferation and differentiation in some tissues. Bach et al. (1995) suggested that IGF-II stimulates the proliferation and differentiation of rat myoblasts. Besides, these pathways are highly correlated with cell proliferation, apoptosis, and differentiation (Sun et al., 2015; Wang et al., 2018). In our study, IGF-I was enriched in the GO terms of positive regulation of phosphatidylinositol 3-kinase signaling and regulation of multicellular organism growth, and IGF-II was enriched in the GO terms of positive regulation of MAPK cascade. Hence, IGF-I and IGF-II may affect the proliferation and differentiation of rumen cells.

Rumen and its microbial abundance are closely related to the growth traits of ruminants (Andersen et al., 2021). Large amounts of VFAs and microbial proteins were produced in rumen with the assistance of rumen microbes (Krause et al., 2020; Newbold and Ramos-Morales 2020; Chai et al., 2021), which are important sources of energy, fatty acids, and amino acids for ruminants (Bergman 1990; Pathak 2008; Li et al., 2013; Liu et al., 2018). Fatty acids are associated with the deposition of marbling and backfat (Smith and Johnson 2015; Ueda et al., 2019; Zhang et al., 2019). Amino acids are the building blocks of proteins that are necessary for body protein synthesis such as muscle growth or milk protein secretion (Abdoun et al., 2006; Wu 2014). Previous research reported that *SORT1*, *ITGA6*, and *TMEM39B* were associated with intramuscular fat deposition (Cesar et al., 2018; Hérault et al., 2018; Ueda et al., 2021). Chromosome locations of *PAQR3* co-located with QTL associated with fat deposition and one missense variant likely affect intramuscular fat *via* the *LNPEP* gene (Pena et al., 2013; Derks et al., 2021). We integrated differential genes obtained from the transcriptome with GWAS data using sum-based marker-set test method, which have been shown to be more potent or at least equivalent to most commonly used marker-set test methods for polygenic traits (Sørensen et al., 2017; Cai et al., 2021). In birth vs. youth comparison, the GWAS signals of MS trait were significantly enriched in fold change 0∼1 upregulated genes, which implied that these slightly upregulated genes were related to beef quality. Interestingly, we found that GWAS signals of SW trait were significantly enriched in fold change >3 downregulated genes, indicating that these dramatically downregulated genes may be correlated with weight of stomach. Furthermore, *SORT1*, *ITGA6*, *TMEM39B*, *PAQR3*, and *LNPEP* belong to fold change 0∼1 upregulated genes, and *IGF2* belongs to fold change >3 downregulated genes. We can make a hypothesis that *SORT1*, *ITGA6*, *TMEM39B*, *PAQR3*, and *LNPEP* may be related to MS trait and *IGF2* related to SW, which needs to be verified in future study. In birth vs. adult comparison, GWAS signals for CW, LMW, REA, and BFT were significantly enriched in fold change 0∼1 up/downregulated genes (Figures 5C,D). This implied that these slightly upregulated genes may be associated with CW, LMW, REA, and BFT traits. Many genes were reported to be associated with CW in previous studies. Fu et al. (2020) reported that a novel 65-bp indel in the *GOLGB1* gene is related to chicken growth and body weight. *PLA2R1* was associated with fat deposition and body weight, and the haplotype of *ACVR2A* gene had a significant effect on CW (Gheyas et al., 2015; Bhattacharya et al., 2016). Moreover, *BMP7* and *NFIA* are associated with hot CW and LMW traits in beef cattle (Wang et al., 2020). *TWIST2* and *ACSL1* genes may be related to REA trait (Carvalho et al., 2019; Silva-Vignato et al., 2019). Positive correlations were found between *FBP1* and *PCCA* gene expressions with BFT (Fassah et al., 2018). In addition, *PECR* and *ACAT1* genes may be related to BFT trait (Piórkowska et al., 2017; Silva-Vignato et al., 2019). Moreover, these above 11 genes included in fold change 0∼1 up/downregulated genes group implied that these genes may be involved in regulating economic traits in beef cattle. This need for further investigation. Besides, the differential genes were divided into modules for enrichment analysis. We found that GWAS signals for CW, LMW, and REA were enriched in turquoise module, and GWAS signals for CW were enriched in lightgreen module (Supplementary Figures S8B,C). These implied that turquoise module genes might be related to CW, LMW, and REA traits and lightgreen module genes might be related to CW trait. We also found that *GOLGB1*, *ACVR2A*, and *TWIST2* belong to turquoise module, which was consistent with our above hypothesis that *GOLGB1* and *ACVR2A* might affect CW traits and *TWIST2* affect REA trait.

## Conclusion

This study explored the genes of *CCNB1*, *CCNB2*, *IGF1*, *IGF2*, *HMGCL*, *BDH1*, *ACAT1*, *HMGCS2*, and *CREBBP* on rumen proliferation and development. On the basis of our results, we can make a hypothesis that *SORT1*, *ITGA6*, *TMEM39B*, *PAQR3*, and *LNPEP* may regulate MS trait; *GOLGB1*, *PLA2R1*, and *ACVR2A* may regulate CW trait; *IGF2* may regulate SW trait; *BMP7* and *NFIA* may regulate LMW trait; *TWIST2* and *ACSL1* may regulate REA trait; and *FBP1*, *PCCA*, *PECR*, and *ACAT1* may regulate BFT trait.

## Data Availability

The raw sequence data reported in this paper have been deposited in the Genome Sequence Archive (Genomics, Proteomics & Bioinformatics 2021) in National Genomics Data Center (Nucleic Acids Res 2021), China National Center for Bioinformation/Beijing Institute of Genomics, Chinese Academy of Sciences (GSA: CRA005438) that are publicly accessible at https://ngdc.cncb.ac.cn/gsa. Genotype data have been submitted to Dryad: doi:10.5061/dryad.4qc06.

## References

[B1] Abdoun K. Stumpff F. Martens H. (2006). Ammonia and Urea Transport across the Rumen Epithelium: a Review. Anim. Health Res. Rev. 7, 43–59. 10.1017/s1466252307001156 17389053

[B2] Andersen T. O. Kunath B. J. Hagen L. H. Arntzen M. Ø. Pope P. B. (2021). Rumen Metaproteomics: Closer to Linking Rumen Microbial Function to Animal Productivity Traits. Methods 186, 42–51. 10.1016/j.ymeth.2020.07.011 32758682

[B3] Bach L. A. Salemi R. Leeding K. S. (1995). Roles of Insulin-like Growth Factor (IGF) Receptors and IGF-Binding Proteins in IGF-II-Induced Proliferation and Differentiation of L6A1 Rat Myoblasts. Endocrinology 136, 5061–5069. 10.1210/endo.136.11.7588242 7588242

[B4] Baldwin R. L. Wu S. Li W. Li C. Bequette B. J. Li R. W. (2012). Quantification of Transcriptome Responses of the Rumen Epithelium to Butyrate Infusion Using RNA-Seq Technology. Gene Regul. Syst. Bio 6, 67–80. 10.4137/GRSB.S9687 PMC3362330 22654504

[B5] Baldwin R. L. (1999). The Proliferative Actions of Insulin, Insulin-like Growth Factor-I, Epidermal Growth Factor, Butyrate and Propionate on Ruminal Epithelial Cells *In Vitro* . Small Ruminant Res. 32, 261–268. 10.1016/s0921-4488(98)00188-6

[B6] Benesch F. Dengler F. Masur F. Pfannkuche H. Gäbel G. (2014). Monocarboxylate Transporters 1 and 4: Expression and Regulation by PPARα in Ovine Ruminal Epithelial Cells. Am. J. Physiology-Regulatory, Integr. Comp. Physiol. 307, R1428–R1437. 10.1152/ajpregu.00408.2013 25320343

[B7] Bergman E. N. (1990). Energy Contributions of Volatile Fatty Acids from the Gastrointestinal Tract in Various Species. Physiol. Rev. 70, 567–590. 10.1152/physrev.1990.70.2.567 2181501

[B8] Bhattacharya T. K. Chatterjee R. N. Dushyanth K. Paswan C. Guru Vishnu P. (2016). Activin Receptor 2A and Activin Receptor 2B Genes in Chicken: Effect on Carcass Traits. J. Appl. Anim. Res. 44, 480–486. 10.1080/09712119.2015.1091321

[B9] Cai W. Li C. Li J. Song J. Zhang S. (2021). Integrated Small RNA Sequencing, Transcriptome and GWAS Data Reveal microRNA Regulation in Response to Milk Protein Traits in Chinese Holstein Cattle. Front. Genet. 12. 10.3389/fgene.2021.726706 PMC8546187 34712266

[B10] Carvalho M. Baldi F. Alexandre P. Santana M. Ventura R. (2019). Genomic Regions and Genes Associated with Carcass Quality in Nelore Cattle. Genet. Mol. Res. GMR 18, gmr18226. 10.4238/gmr18226

[B11] Cesar A. S. M. Regitano L. C. A. Koltes J. E. Fritz-Waters E. R. Lanna D. P. D. Gasparin G. (2015). Putative Regulatory Factors Associated with Intramuscular Fat Content. PLoS One 10, e0128350. 10.1371/journal.pone.0128350 26042666 PMC4456163

[B12] Cesar A. S. M. Regitano L. C. A. Reecy J. M. Poleti M. D. Oliveira P. S. N. de Oliveira G. B. (2018). Identification of Putative Regulatory Regions and Transcription Factors Associated with Intramuscular Fat Content Traits. BMC Genomics 19, 499. 10.1186/s12864-018-4871-y 29945546 PMC6020320

[B13] Chai J. Lv X. Diao Q. Usdrowski H. Zhuang Y. (2021). Solid Diet Manipulates Rumen Epithelial Microbiota and its Interactions with Host Transcriptomic in Young Ruminants. Environ. Microbiol. n/a. 10.1111/1462-2920.15757 PMC9292864 34490978

[B14] Connor E. E. Baldwin R. L. Walker M. P. Ellis S. E. Li C. Kahl S. (2014). Transcriptional Regulators Transforming Growth Factor-Β1 and Estrogen-Related Receptor-α Identified as Putative Mediators of Calf Rumen Epithelial Tissue Development and Function during Weaning. J. Dairy Sci. 97, 4193–4207. 10.3168/jds.2013-7471 24767884

[B15] Connor E. E. Baldwin R. L. Li C.-j. Li R. W. Chung H. (2013). Gene Expression in Bovine Rumen Epithelium during Weaning Identifies Molecular Regulators of Rumen Development and Growth. Funct. Integr. Genomics 13, 133–142. 10.1007/s10142-012-0308-x 23314861

[B16] Coverdale J. A. Tyler H. D. Quigley J. D. 3rd Brumm J. A. (2004). Effect of Various Levels of Forage and Form of Diet on Rumen Development and Growth in Calves. J. Dairy Sci. 87, 2554–2562. 10.3168/jds.s0022-0302(04)73380-9 15328279

[B17] Cui C. Li L. Zhen J. (2018). Bioinformatic Analysis Reveals the Key Pathways and Genes in Early-Onset Breast Cancer. Med. Oncol. 35, 67. 10.1007/s12032-018-1130-7 29644522

[B18] Čunátová K. Reguera D. P. Houštěk J. Mráček T. Pecina P. (2020). Role of Cytochrome C Oxidase Nuclear-Encoded Subunits in Health and Disease. Physiol. Res. 69, 947–965. 33129245 10.33549/physiolres.934446 PMC8549878

[B19] Derks M. F. L. Gross C. Lopes M. S. Reinders M. J. T. Bosse M. Gjuvsland A. B. (2021). Accelerated Discovery of Functional Genomic Variation in Pigs. Genomics 113, 2229–2239. 10.1016/j.ygeno.2021.05.017 34022350

[B20] Du Y.-J. Luo X.-Y. Hao Y.-Z. Zhang T. Hou W.-R. (2007). cDNA Cloning and Overexpression of Acidic Ribosomal Phosphoprotein P1 Gene (RPLP1) from the Giant Panda. Int. J. Biol. Sci. 3, 428–433. 10.7150/ijbs.3.428 18071584 PMC2043164

[B21] Fassah D. M. Jeong J. Y. Baik M. (2018). Hepatic Transcriptional Changes in Critical Genes for Gluconeogenesis Following Castration of Bulls. Asian-australas J. Anim. Sci. 31, 537–547. 10.5713/ajas.17.0875 29502393 PMC5838326

[B22] Fouts D. E. Szpakowski S. Purushe J. Torralba M. Waterman R. C. MacNeil M. D. (2012). Next Generation Sequencing to Define Prokaryotic and Fungal Diversity in the Bovine Rumen. PloS one 7, e48289. 10.1371/journal.pone.0048289 23144861 PMC3492333

[B23] Fritz G. Botelho H. M. Morozova-Roche L. A. Gomes C. M. (2010). Natural and Amyloid Self-Assembly of S100 Proteins: Structural Basis of Functional Diversity. FEBS J. 277, 4578–4590. 10.1111/j.1742-4658.2010.07887.x 20977662

[B24] Fu R. Ren T. Li W. Liang J. Mo G. (2020). A Novel 65-bp Indel in the GOLGB1 Gene Is Associated with Chicken Growth and Carcass Traits, Animals (Basel) 10, 475. 10.3390/ani10030475 PMC7142648 32178328

[B25] Gheyas A. A. Boschiero C. Eory L. Ralph H. Kuo R. Woolliams J. A. (2015). Functional Classification of 15 Million SNPs Detected from Diverse Chicken Populations. DNA Res. 22, 205–217. 10.1093/dnares/dsv005 25926514 PMC4463845

[B26] Gui H. Shen Z. (2016). Concentrate Diet Modulation of Ruminal Genes Involved in Cell Proliferation and Apoptosis Is Related to Combined Effects of Short-Chain Fatty Acid and pH in Rumen of Goats. J. Dairy Sci. 99, 6627–6638. 10.3168/jds.2015-10446 27236768

[B27] Guo S. Quan S. Zou S. (2021). Roles of the Notch Signaling Pathway in Ovarian Functioning. Reprod. Sci. 28, 2770–2778. 10.1007/s43032-021-00610-6 34008156

[B28] Hérault F. Damon M. Cherel P. Le Roy P. (2018). Combined GWAS and LDLA Approaches to Improve Genome-wide Quantitative Trait Loci Detection Affecting Carcass and Meat Quality Traits in Pig. Meat Sci. 135, 148–158. 10.1016/j.meatsci.2017.09.015 29035812

[B29] Huang D. W. Sherman B. T. Tan Q. Kir J. Liu D. Bryant D. (2007). DAVID Bioinformatics Resources: Expanded Annotation Database and Novel Algorithms to Better Extract Biology from Large Gene Lists. Nucleic Acids Res. 35, W169–W175. 10.1093/nar/gkm415 17576678 PMC1933169

[B30] Huang S. S. Huang J. S. (2005). TGF-β Control of Cell Proliferation. J. Cel. Biochem. 96, 447–462. 10.1002/jcb.20558 16088940

[B31] Huang W. Guo Y. Du W. Zhang X. Li A. Miao X. (2017). Global Transcriptome Analysis Identifies Differentially Expressed Genes Related to Lipid Metabolism in Wagyu and Holstein Cattle. Sci. Rep. 7, 5278. 10.1038/s41598-017-05702-5 28706200 PMC5509646

[B32] Jami E. White B. A. Mizrahi I. (2014). “Potential Role of the Bovine Rumen Microbiome in Modulating Milk Composition and Feed Efficiency, PLoS ONE, 9, e85423. 10.1371/journal.pone.0085423 24465556 PMC3899005

[B33] Kabiri Z. Greicius G. Zaribafzadeh H. Hemmerich A. Counter C. M. Virshup D. M. (2018). Wnt Signaling Suppresses MAPK-Driven Proliferation of Intestinal Stem Cells. J. Clin. Invest. 128, 3806–3812. 10.1172/jci99325 30059017 PMC6118584

[B34] Kalkhoven E. (2004). CBP and P300: HATs for Different Occasions. Biochem. Pharmacol. 68, 1145–1155. 10.1016/j.bcp.2004.03.045 15313412

[B35] Kato D. Suzuki Y. Haga S. So K. Yamauchi E. Nakano M. (2016). Utilization of Digital Differential Display to Identify Differentially Expressed Genes Related to Rumen Development. Anim. Sci. J. 87, 584–590. 10.1111/asj.12448 26388291

[B36] Kim D. Shin H. Joung J. G. Lee S. Y. Kim J. H. (2013). Intra-relation Reconstruction from Inter-relation: miRNA to Gene Expression. BMC Syst. Biol. 7 Suppl 3 (Suppl. 3), S8. 10.1186/1752-0509-7-S3-S8 PMC3852212 24521265

[B37] Kim D. Langmead B. Salzberg S. L. (2015). HISAT: a Fast Spliced Aligner with Low Memory Requirements. Nat. Methods 12, 357–360. 10.1038/nmeth.3317 25751142 PMC4655817

[B38] Kim M. Park T. Jeong J. Y. Baek Y. Lee H. J. (2020). Association between Rumen Microbiota and Marbling Score in Korean Native Beef Cattle, Animals (Basel) 10, 712. 10.3390/ani10040712 PMC7222830 32325868

[B39] Kong L. Liu G. Deng M. Lian Z. Han Y. Sun B. (2020). Growth Retardation-Responsive Analysis of mRNAs and Long Noncoding RNAs in the Liver Tissue of Leiqiong Cattle. Sci. Rep. 10, 14254. 10.1038/s41598-020-71206-4 32868811 PMC7459292

[B40] Kostiuk M. A. Keller B. O. Berthiaume L. G. (2010). Palmitoylation of Ketogenic Enzyme HMGCS2 Enhances its Interaction with PPARα and Transcription at theHmgcs2PPRE. FASEB j. 24, 1914–1924. 10.1096/fj.09-149765 20124434 PMC2874477

[B41] Krause T. R. Lourenco J. M. Welch C. B. Rothrock M. J. Callaway T. R. Pringle T. D. (2020). The Relationship between the Rumen Microbiome and Carcass merit in Angus Steers. J. Anim. Sci. 98. 10.1093/jas/skaa287 PMC7526868 32877916

[B42] Kristensen N. B. Sehested J. Jensen S. K. Vestergaard M. (2007). Effect of Milk Allowance on Concentrate Intake, Ruminal Environment, and Ruminal Development in Milk-Fed Holstein Calves. J. Dairy Sci. 90, 4346–4355. 10.3168/jds.2006-885 17699055

[B43] Lane M. A. Baldwin R. L. Jesse B. W. (2002). Developmental Changes in Ketogenic Enzyme Gene Expression during Sheep Rumen Development1. J. Anim. Sci. 80, 1538–1544. 10.2527/2002.8061538x 12078735

[B44] Langfelder P. Horvath S. (2008). WGCNA: an R Package for Weighted Correlation Network Analysis. BMC Bioinformatics 9, 559. 10.1186/1471-2105-9-559 19114008 PMC2631488

[B45] Langfelder P. Zhang B. Horvath S. (2008). Defining Clusters from a Hierarchical Cluster Tree: the Dynamic Tree Cut Package for R. Bioinformatics 24, 719–720. 10.1093/bioinformatics/btm563 18024473

[B46] Li C. Cai W. Zhou C. Yin H. Zhang Z. Loor J. J. (2016). RNA-seq Reveals 10 Novel Promising Candidate Genes Affecting Milk Protein Concentration in the Chinese Holstein Population, Scientific Rep., 6, 26813. 10.1038/srep26813 PMC4890585 27254118

[B47] Li H. Handsaker B. Wysoker A. Fennell T. Ruan J. Homer N. (2009). The Sequence Alignment/Map Format and SAMtools. Bioinformatics 25, 2078–2079. 10.1093/bioinformatics/btp352 19505943 PMC2723002

[B48] Li W. Edwards A. Riehle C. Cox M. S. Raabis S. Skarlupka J. H. (2019). Transcriptomics Analysis of Host Liver and Meta-Transcriptome Analysis of Rumen Epimural Microbial Community in Young Calves Treated with Artificial Dosing of Rumen Content from Adult Donor Cow. Sci. Rep. 9, 790. 10.1038/s41598-018-37033-4 30692556 PMC6349911

[B49] Li X. Chen H. Guan Y. Li X. Lei L. Liu J. (2013). Acetic Acid Activates the AMP-Activated Protein Kinase Signaling Pathway to Regulate Lipid Metabolism in Bovine Hepatocytes. PLoS One 8, e67880. 10.1371/journal.pone.0067880 23861826 PMC3701595

[B50] Liao Y. Smyth G. K. Shi W. (2014). featureCounts: an Efficient General Purpose Program for Assigning Sequence Reads to Genomic Features. Bioinformatics 30, 923–930. 10.1093/bioinformatics/btt656 24227677

[B51] Lin L. Xie F. Sun D. Liu J. Zhu W. Mao S. (2019). Ruminal Microbiome-Host Crosstalk Stimulates the Development of the Ruminal Epithelium in a Lamb Model. Microbiome 7, 83. 10.1186/s40168-019-0701-y 31159860 PMC6547527

[B52] Lin S. Fang L. Kang X. Liu S. Liu M. Connor E. E. (2020). Establishment and Transcriptomic Analyses of a Cattle Rumen Epithelial Primary Cells (REPC) Culture by Bulk and Single-Cell RNA Sequencing to Elucidate Interactions of Butyrate and Rumen Development. Heliyon 6, e04112. 10.1016/j.heliyon.2020.e04112 32551379 PMC7287249

[B53] Liu J. Li X. Dong G.-L. Zhang H.-W. Chen D.-L. Du J.-J. (2008). In Silico analysis and Verification of S100 Gene Expression in Gastric Cancer. BMC Cancer 8, 261. 10.1186/1471-2407-8-261 18793447 PMC2567992

[B54] Liu Q. Wang C. Guo G. Huo W. J. Zhang Y. L. Pei C. X. (2018). Effects of Branched-Chain Volatile Fatty Acids Supplementation on Growth Performance, Ruminal Fermentation, Nutrient Digestibility, Hepatic Lipid Content and Gene Expression of Dairy Calves. Anim. Feed Sci. Technol. 237, 27–34. 10.1016/j.anifeedsci.2018.01.006

[B55] Love M. I. Huber W. Anders S. (2014). Moderated Estimation of Fold Change and Dispersion for RNA-Seq Data with DESeq2. Genome Biol. 15, 550. 10.1186/s13059-014-0550-8 25516281 PMC4302049

[B56] Malmuthuge N. Liang G. Guan L. L. (2019). Regulation of Rumen Development in Neonatal Ruminants through Microbial Metagenomes and Host Transcriptomes. Genome Biol. 20, 172. 10.1186/s13059-019-1786-0 31443695 PMC6708143

[B57] Naeem A. Drackley J. K. Stamey J. Loor J. J. (2012). Role of Metabolic and Cellular Proliferation Genes in Ruminal Development in Response to Enhanced Plane of Nutrition in Neonatal Holstein Calves. J. Dairy Sci. 95, 1807–1820. 10.3168/jds.2011-4709 22459829

[B58] Newbold C. J. Ramos-Morales E. (2020). Review: Ruminal Microbiome and Microbial Metabolome: Effects of Diet and Ruminant Host. Animal 14, s78–s86. 10.1017/s1751731119003252 32024572

[B59] Nishihara K. Suzuki Y. Roh S. (2020). Ruminal Epithelial Insulin-like Growth Factor-Binding Proteins 2, 3, and 6 Are Associated with Epithelial Cell Proliferation. Anim. Sci. J. 91, e13422. 10.1111/asj.13422 32648312

[B60] Norouzian M. A. Valizadeh R. Vahmani P. (2011). Rumen Development and Growth of Balouchi Lambs Offered Alfalfa hay Pre- and post-weaning. Trop. Anim. Health Prod. 43, 1169–1174. 10.1007/s11250-011-9819-z 21465104

[B61] Pathak A. (2008). Various Factors Affecting Microbial Protein Synthesis in the Rumen. Veterinary World 1 (6), 186–189.

[B62] Pena R. N. Noguera J. L. Casellas J. Díaz I. Fernández A. I. Folch J. M. (2013). Transcriptional Analysis of Intramuscular Fatty Acid Composition in the Longissimus Thoracis Muscle of Iberian × Landrace Back-Crossed Pigs. Anim. Genet. 44, 648–660. 10.1111/age.12066 23826865

[B63] Pertea M. Pertea G. M. Antonescu C. M. Chang T.-C. Mendell J. T. Salzberg S. L. (2015). StringTie Enables Improved Reconstruction of a Transcriptome from RNA-Seq Reads. Nat. Biotechnol. 33, 290–295. 10.1038/nbt.3122 25690850 PMC4643835

[B64] Piórkowska K. Tyra M. Ropka-Molik K. Podbielska A. (2017). Evolution of Peroxisomal Trans-2-enoyl-CoA Reductase ( PECR ) as Candidate Gene for Meat Quality. Livestock Sci. 201, 85–91. 10.1016/j.livsci.2017.05.004

[B65] Poulsen M. Jensen B. B. Engberg R. M. (2012). The Effect of Pectin, Corn and Wheat Starch, Inulin and pH on *In Vitro* Production of Methane, Short Chain Fatty Acids and on the Microbial Community Composition in Rumen Fluid. Anaerobe 18, 83–90. 10.1016/j.anaerobe.2011.12.009 22193552

[B66] Purcell S. Neale B. Todd-Brown K. Thomas L. Ferreira M. A. R. Bender D. (2007). PLINK: a Tool Set for Whole-Genome Association and Population-Based Linkage Analyses. Am. J. Hum. Genet. 81, 559–575. 10.1086/519795 17701901 PMC1950838

[B67] Quigley J. D. 3rd Schwab C. G. Hylton W. E. (1985). Development of Rumen Function in Calves: Nature of Protein Reaching the Abomasum. J. Dairy Sci. 68, 694–702. 10.3168/jds.s0022-0302(85)80875-4 3989088

[B68] Reddy K. E. Jeong J. Baek Y.-C. Oh Y. K. Kim M. So K. M. (2017). Early Weaning of Calves after Different Dietary Regimens Affects Later Rumen Development, Growth, and Carcass Traits in Hanwoo Cattle. Asian-australas J. Anim. Sci. 30, 1425–1434. 10.5713/ajas.17.0315 28728406 PMC5582327

[B69] Rey M. Enjalbert F. Combes S. Cauquil L. Bouchez O. Monteils V. (2014). Establishment of Ruminal Bacterial Community in Dairy Calves from Birth to Weaning Is Sequential. J. Appl. Microbiol. 116, 245–257. 10.1111/jam.12405 24279326

[B70] Rey M. Enjalbert F. Monteils V. (2012). Establishment of Ruminal Enzyme Activities and Fermentation Capacity in Dairy Calves from Birth through Weaning. J. Dairy Sci. 95, 1500–1512. 10.3168/jds.2011-4902 22365231

[B71] Reynolds C. K. Dürst B. Lupoli B. Humphries D. J. Beever D. E. (2004). Visceral Tissue Mass and Rumen Volume in Dairy Cows during the Transition from Late Gestation to Early Lactation. J. Dairy Sci. 87, 961–971. 10.3168/jds.s0022-0302(04)73240-3 15259230

[B72] Sakata T. Tamate H. (1978). Rumen Epithelial Cell Proliferation Accelerated by Rapid Increase in Intraruminal Butyrate. J. Dairy Sci. 61, 1109–1113. 10.3168/jds.s0022-0302(78)83694-7 721981

[B73] Seddigh S. Darabi M. (2018). Functional, Structural, and Phylogenetic Analysis of Mitochondrial Cytochrome B (Cytb) in Insects. Mitochondrial DNA A 29, 236–249. 10.1080/24701394.2016.1275596 28116966

[B74] Sehested J. Diernæs L. Møller P. D. Skadhauge E. (1999). Ruminal Transport and Metabolism of Short-Chain Fatty Acids (SCFA) *In Vitro*: Effect of SCFA Chain Length and pH. Comp. Biochem. Physiol. A: Mol. Integr. Physiol. 123, 359–368. 10.1016/s1095-6433(99)00074-4 10581701

[B75] Shabat S. K. B. Sasson G. Doron-Faigenboim A. Durman T. Yaacoby S. Berg Miller M. E. (2016). Specific Microbiome-dependent Mechanisms Underlie the Energy Harvest Efficiency of Ruminants. Isme J. 10, 2958–2972. 10.1038/ismej.2016.62 27152936 PMC5148187

[B76] Shannon P. Markiel A. Ozier O. Baliga N. S. Wang J. T. Ramage D. (2003). Cytoscape: a Software Environment for Integrated Models of Biomolecular Interaction Networks. Genome Res. 13, 2498–2504. 10.1101/gr.1239303 14597658 PMC403769

[B77] Silva-Vignato B. Coutinho L. L. Poleti M. D. Cesar A. S. M. Moncau C. T. Regitano L. C. A. (2019). Gene Co-expression Networks Associated with Carcass Traits Reveal New Pathways for Muscle and Fat Deposition in Nelore Cattle. BMC Genomics 20, 32. 10.1186/s12864-018-5345-y 30630417 PMC6329100

[B78] Smith S. B. Johnson B. J. (2016). 0794 Marbling: Management of Cattle to Maximize the Deposition of Intramuscular Adipose Tissue. Journal of Animal Science 94, 382.

[B79] Sørensen I. F. Edwards S. M. Rohde P. D. Sørensen P. (2017). Multiple Trait Covariance Association Test Identifies Gene Ontology Categories Associated with Chill Coma Recovery Time in *Drosophila melanogaster* . Sci. Rep. 7, 2413. 28546557 10.1038/s41598-017-02281-3 PMC5445101

[B80] Sun D. Yin Y. Guo C. Liu L. Mao S. Zhu W. (2021). Transcriptomic Analysis Reveals the Molecular Mechanisms of Rumen wall Morphological and Functional Development Induced by Different Solid Diet Introduction in a Lamb Model. J. Anim. Sci Biotechnol 12, 33. 10.1186/s40104-021-00556-4 33750470 PMC7944623

[B81] Sun Y. Liu W.-Z. Liu T. Feng X. Yang N. Zhou H.-F. (2015). Signaling Pathway of MAPK/ERK in Cell Proliferation, Differentiation, Migration, Senescence and Apoptosis. J. Receptors Signal Transduction 35, 600–604. 10.3109/10799893.2015.1030412 26096166

[B82] Szklarczyk D. Franceschini A. Wyder S. Forslund K. Heller D. Huerta-Cepas J. (2015). STRING V10: Protein-Protein Interaction Networks, Integrated over the Tree of Life. Nucleic Acids Res. 43, D447–D452. 10.1093/nar/gku1003 25352553 PMC4383874

[B83] Tamate H. McGilliard A. D. Jacobson N. L. Getty R. (1962). Effect of Various Dietaries on the Anatomical Development of the Stomach in the Calf. J. Dairy Sci. 45, 408–420. 10.3168/jds.s0022-0302(62)89406-5

[B84] Ueda S. Hosoda M. Yoshino K.-I. Yamanoue M. Shirai Y. (2021). Gene Expression Analysis Provides New Insights into the Mechanism of Intramuscular Fat Formation in Japanese Black Cattle. Genes 12, 1107. 10.3390/genes12081107 34440281 PMC8391117

[B85] Ueda S. Iwamoto E. Kato Y. Shinohara M. Shirai Y. Yamanoue M. (2019). Comparative Metabolomics of Japanese Black Cattle Beef and Other Meats Using Gas Chromatography-Mass Spectrometry. Biosci. Biotechnol. Biochem. 83, 137–147. 10.1080/09168451.2018.1528139 30336733

[B86] Wang Y. Zhang F. Mukiibi R. Chen L. Vinsky M. Plastow G. (2020). Genetic Architecture of Quantitative Traits in Beef Cattle Revealed by Genome Wide Association Studies of Imputed Whole Genome Sequence Variants: II: Carcass merit Traits. BMC Genomics 21, 38. 10.1186/s12864-019-6273-1 31931697 PMC6958779

[B87] Wang Y. Zhao H. Shao Y. Liu J. Li J. Xing M. (2018). Interplay between Elemental Imbalance-Related PI3K/Akt/mTOR-Regulated Apoptosis and Autophagy in Arsenic (III)-induced Jejunum Toxicity of Chicken. Environ. Sci. Pollut. Res. 25, 18662–18672. 10.1007/s11356-018-2059-2 29705899

[B88] Warner R. G. Flatt W. P. Loosli J. K. (1956). Ruminant Nutrition, Dietary Factors Influencing Development of Ruminant Stomach. J. Agric. Food Chem. 4, 788–792. 10.1021/jf60067a003

[B89] Wu G. (2014). Dietary Requirements of Synthesizable Amino Acids by Animals: a Paradigm Shift in Protein Nutrition. J. Anim. Sci Biotechnol 5, 34. 10.1186/2049-1891-5-34 24999386 PMC4082180

[B90] Xiang R. Oddy V. H. Archibald A. L. Vercoe P. E. Dalrymple B. P. (2016). Epithelial, Metabolic and Innate Immunity Transcriptomic Signatures Differentiating the Rumen from Other Sheep and Mammalian Gastrointestinal Tract Tissues. PeerJ 4, e1762. 10.7717/peerj.1762 26989612 PMC4793311

[B91] Xie C. Mao X. Huang J. Ding Y. Wu J. Dong S. (2011). KOBAS 2.0: a Web Server for Annotation and Identification of Enriched Pathways and Diseases. Nucleic Acids Res. 39, W316–W322. 10.1093/nar/gkr483 21715386 PMC3125809

[B92] Xu L. Gao N. Wang Z. Xu L. Liu Y. Chen Y. (2020). Incorporating Genome Annotation into Genomic Prediction for Carcass Traits in Chinese Simmental Beef Cattle. Front. Genet. 11, 481. 10.3389/fgene.2020.00481 32499816 PMC7243208

[B93] Yang J. Lee S. H. Goddard M. E. Visscher P. M. (2011). GCTA: a Tool for Genome-wide Complex Trait Analysis. Am. J. Hum. Genet. 88, 76–82. 10.1016/j.ajhg.2010.11.011 21167468 PMC3014363

[B94] Yang J. Zaitlen N. A. Goddard M. E. Visscher P. M. Price A. L. (2014). Advantages and Pitfalls in the Application of Mixed-Model Association Methods. Nat. Genet. 46, 100–106. 10.1038/ng.2876 24473328 PMC3989144

[B95] Yang W. Shen Z. Martens H. (2012). An Energy-Rich Diet Enhances Expression of Na+/H+ Exchanger Isoform 1 and 3 Messenger RNA in Rumen Epithelium of Goat1. J. Anim. Sci. 90, 307–317. 10.2527/jas.2011-3854 21856899

[B96] Zhang T. Zhang X. Han K. Zhang G. Wang J. Xie K. (2017). Analysis of Long Noncoding RNA and mRNA Using RNA Sequencing during the Differentiation of Intramuscular Preadipocytes in Chicken. PLoS One 12, e0172389. 10.1371/journal.pone.0172389 28199418 PMC5310915

[B97] Zhang Y. Zhang J. Gong H. Cui L. Zhang W. Ma J. (2019). Genetic Correlation of Fatty Acid Composition with Growth, Carcass, Fat Deposition and Meat Quality Traits Based on GWAS Data in Six Pig Populations. Meat Sci. 150, 47–55. 10.1016/j.meatsci.2018.12.008 30584983

