# Supplementary material for: Transcriptome Analysis of Bovine Rumen Tissue in Three Developmental Stages
Source: Front Genet. 2022 Mar 3;13:821406. doi: 10.3389/fgene.2022.821406 (PMC8928727; doi:10.3389/fgene.2022.821406)
Supplement: Supplementary file 10 [file DataSheet1.docx]

***Supplementary Material***

# Supplementary Data

The raw sequence data reported in this paper have been deposited in the Genome Sequence Archive (Genomics, Proteomics & Bioinformatics 2021) in National Genomics Data Center (Nucleic Acids Res 2021), China National Center for Bioinformation / Beijing Institute of Genomics, Chinese Academy of Sciences (GSA: CRA007849) that are publicly accessible at [**https://ngdc.cncb.ac.cn/gsa**](https://ngdc.cncb.ac.cn/gsa) *.*Genotype data have been submitted to Dryad: [doi:10.5061/dryad.4qc06](https://datadryad.org/stash/dataset/doi:10.5061/dryad.4qc06).

# Supplementary Figures

**
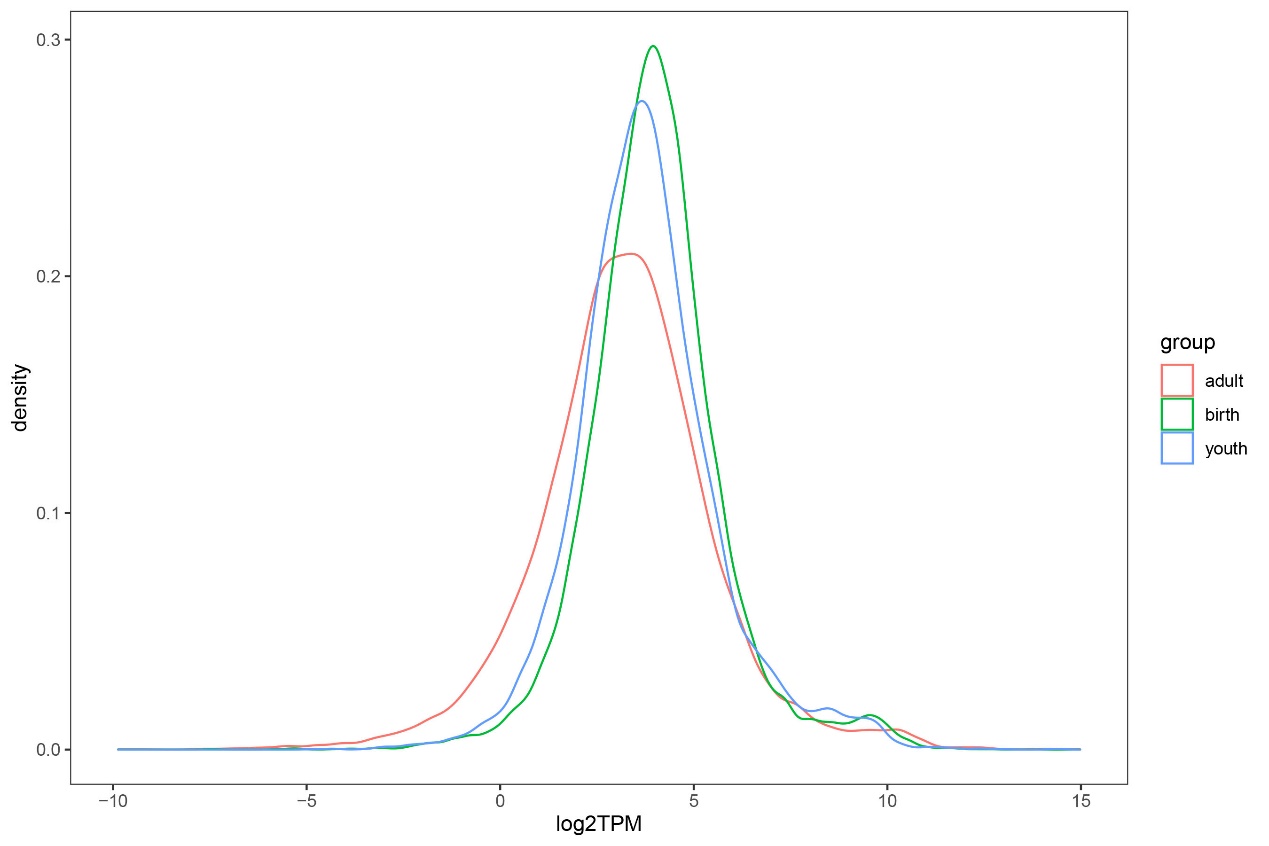
**

**Supplementary Figure 1.** Distribution of all gene expression levels. Red line represents adult gene expression, green line represents birth gene expression and blue line represents youth gene expression.


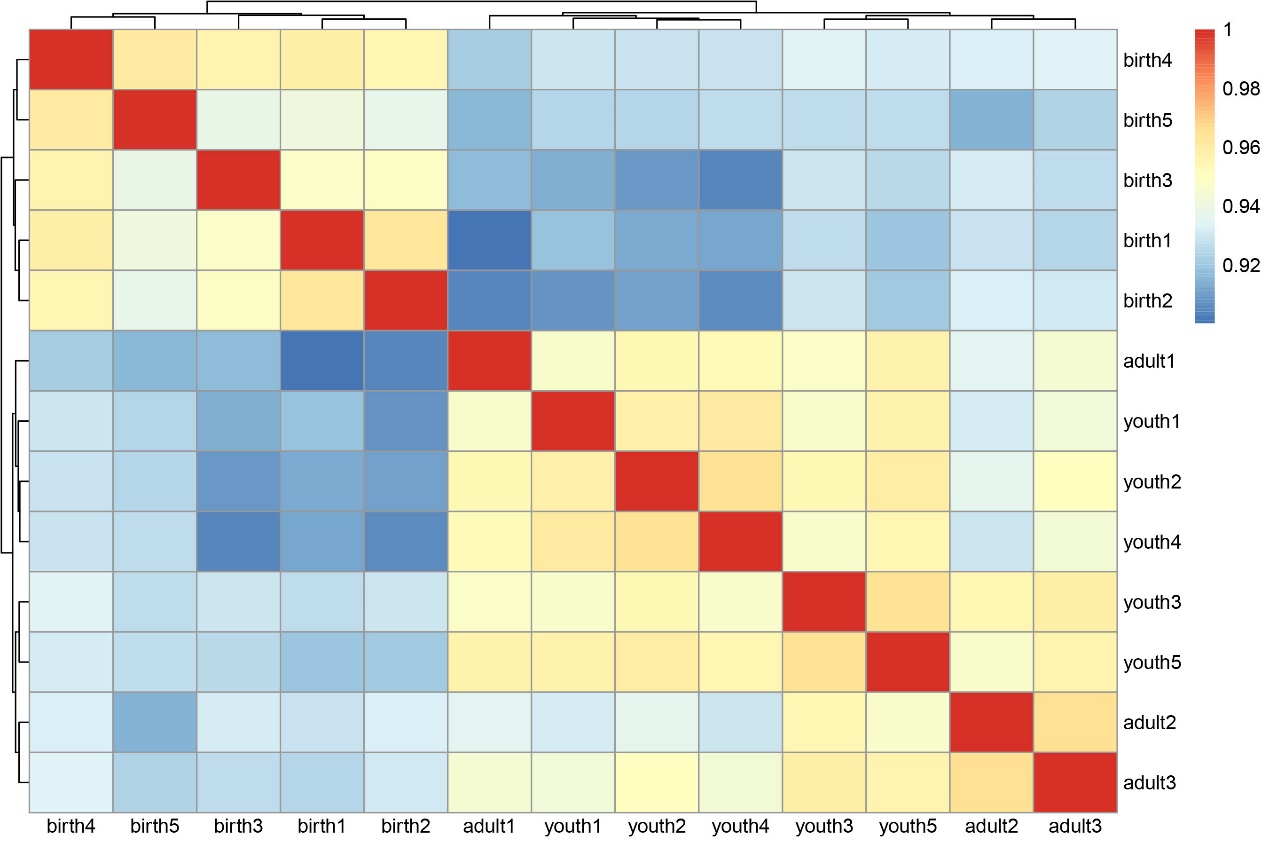


**Supplementary Figure 2.** Correlation of 13 samples. Red indicated higher correlation, and blue showed lower correlation.

**
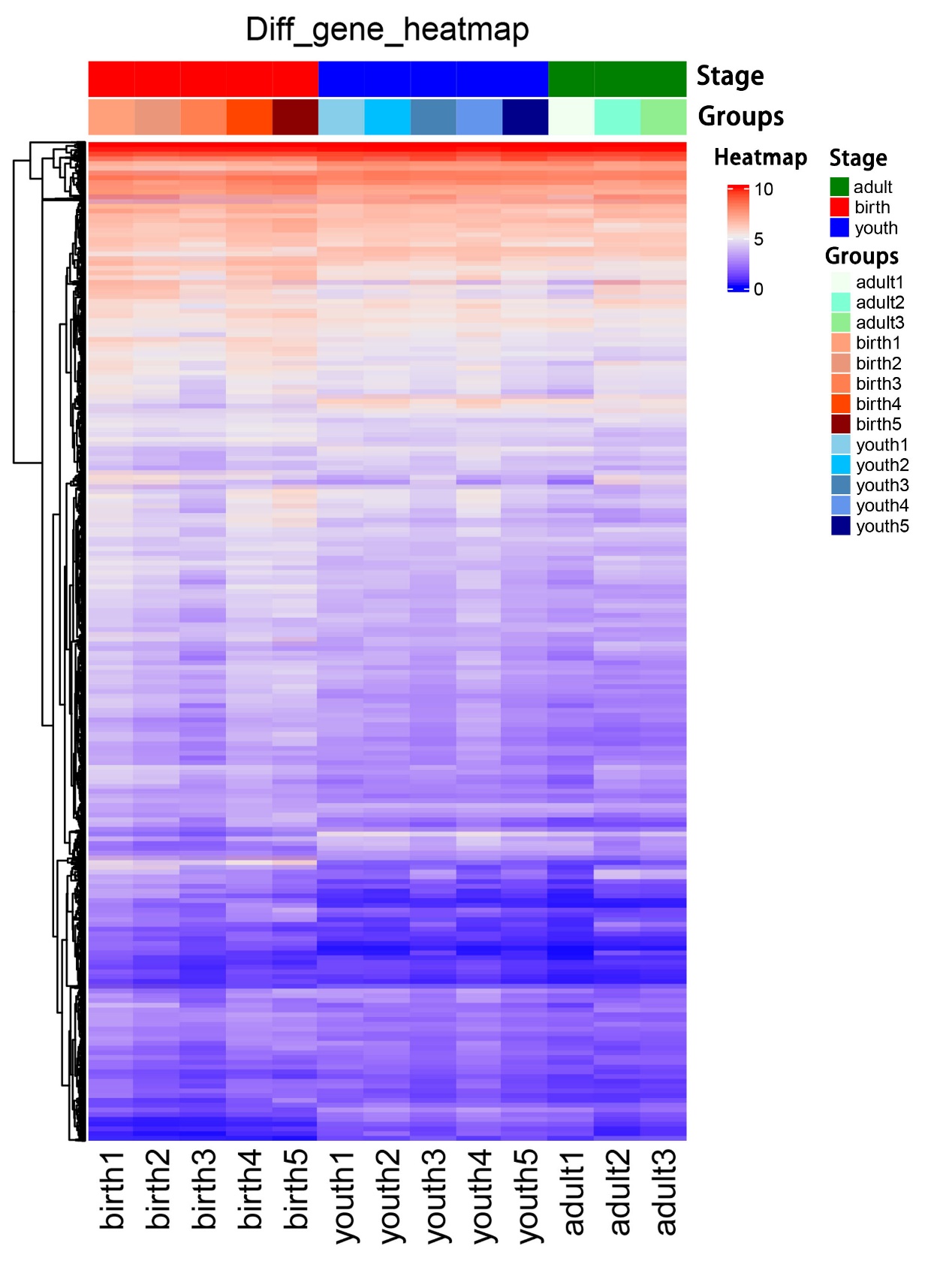
**

**Supplementary Figure 3.** Cluster analysis of all DEGs based on their expression. Red indicated higher expression, and blue showed lower expression.


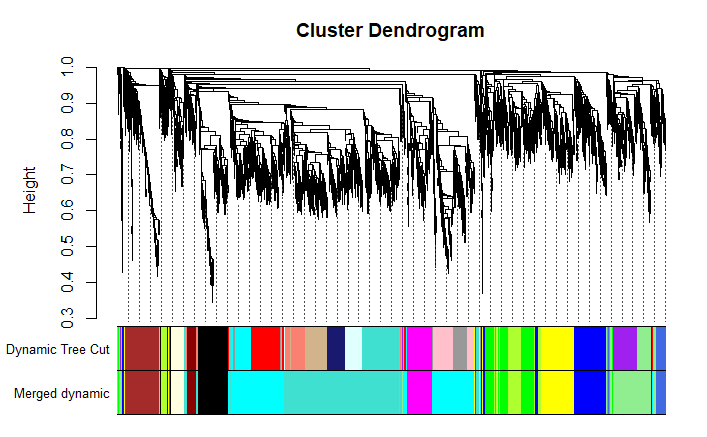


**Supplementary Figure 4.** Hierarchical cluster tree (average linkage, dissTOM) of the 6,048 genes. The color bands provide a simple visual comparison of module assignments (branch cuttings) based on the dynamic hybrid branch cutting method.

**
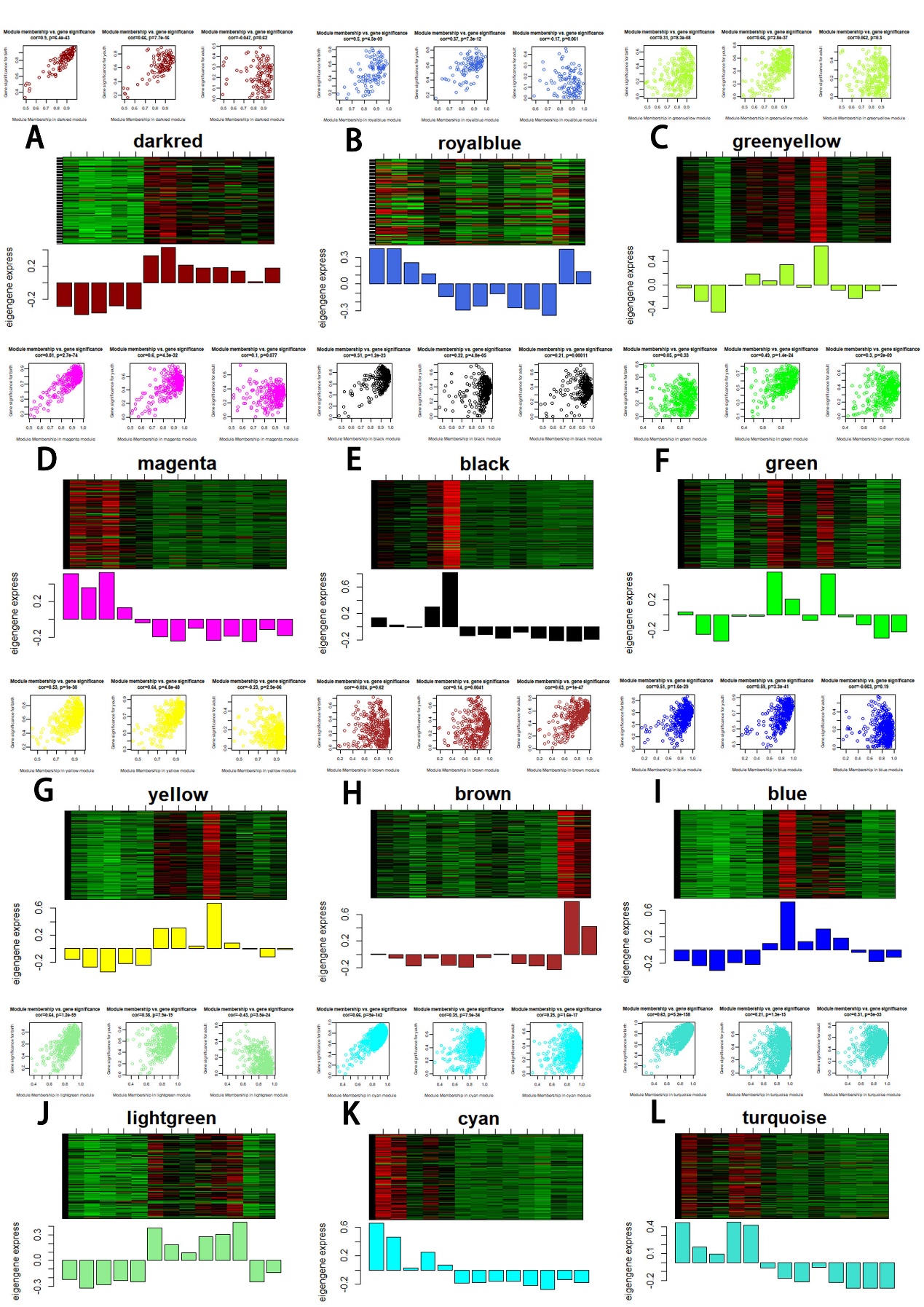
**

**Supplementary Figure 5.** Visualization of GS vs. MM and expression level in stage-specific modules. The scatter plot shows the distribution of the GS and MM of genes in stage-specific modules related to one sample. The P-value represents the significance of a module related to one stage. The heatmap and bar plot correspond to the expression level (ME) of genes in a stage-specific module in the 13 samples (from left to right: birth1, birth2, birth3, birth4, birth5, youth1, youth2, youth3, youth4, youth5, adult1, adult3).

**
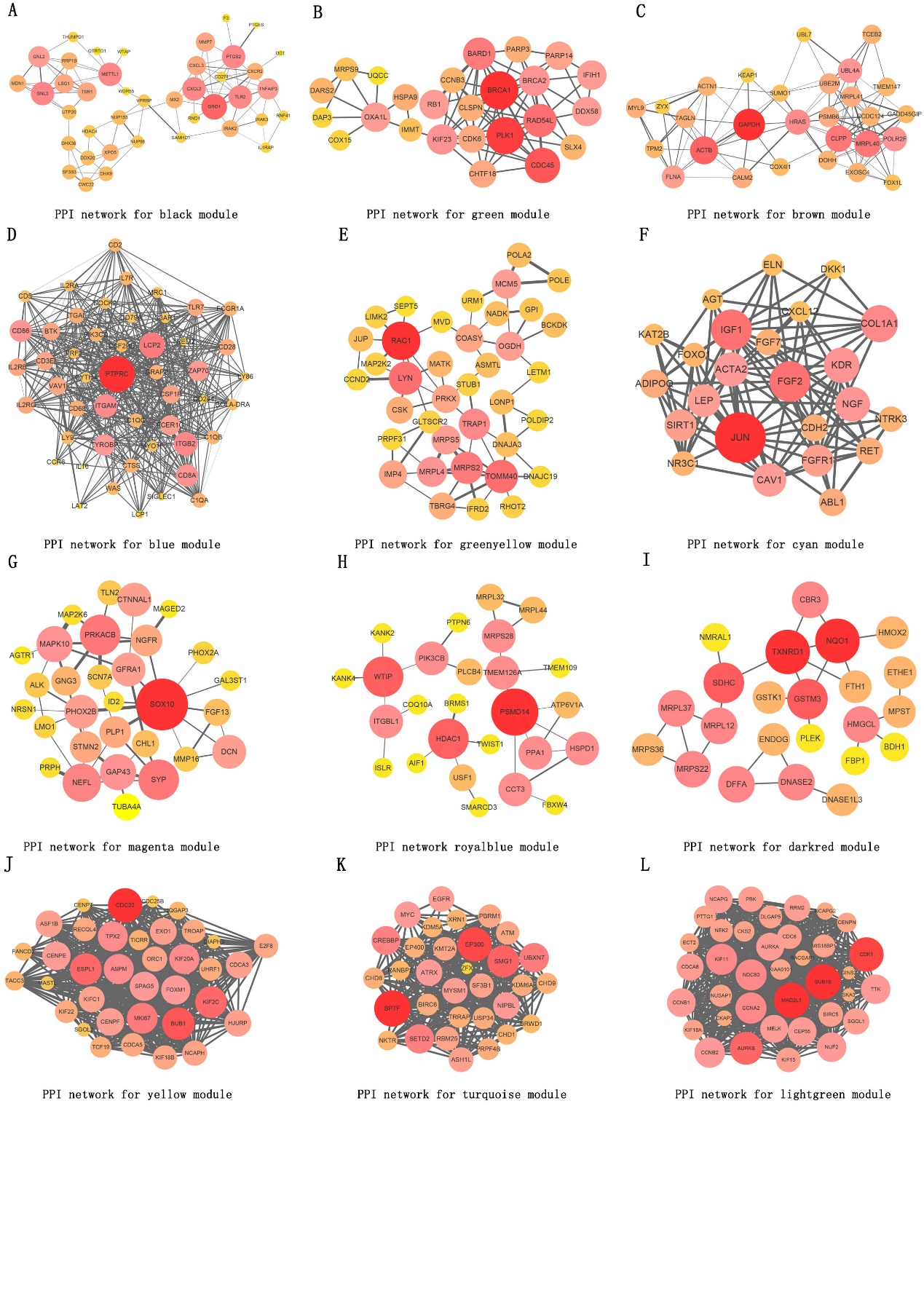
**

**Supplementary Figure 6.** Visualization of potential genes in stage-specific modules. Node size and colors meant different node degree, the width of the edge represented combine score and intramodular connectivity weight, the largest size nodes and red colors meant the highest potential genes.

**
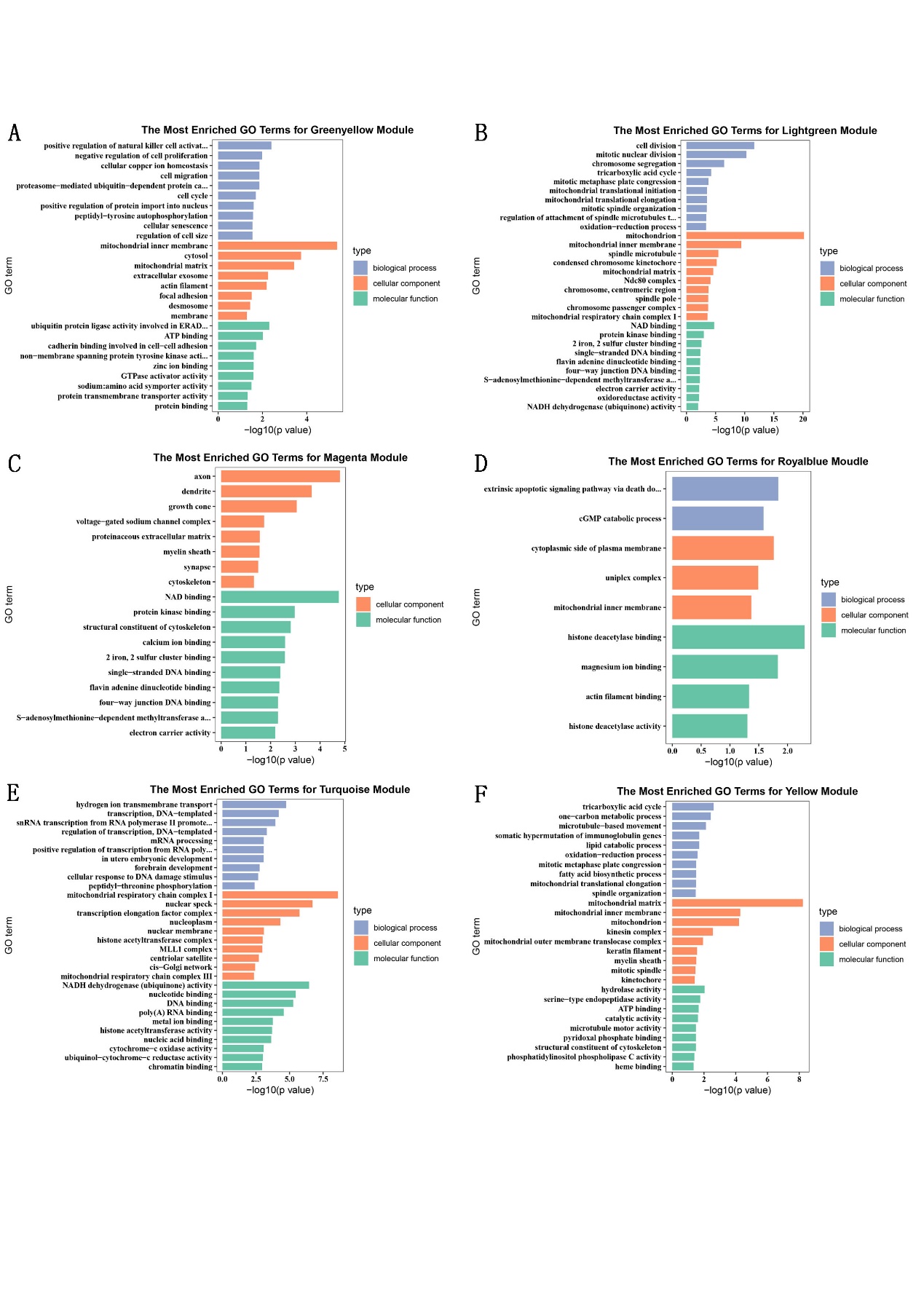
**

**Supplementary Figure 7.** Gene ontology (GO) enrichment analysis of related differentially expressed genes (DEGs). (A) GO enrichment analysis of DEGs in greenyellow module. (B) GO enrichment analysis of DEGs in lightgreen module. (C) GO enrichment analysis of DEGs in magenta module. (D) GO enrichment analysis of DEGs in royalblue module. (E) GO enrichment analysis of DEGs in turquoise module. (F) GO enrichment analysis of DEGs in yellow module.

**
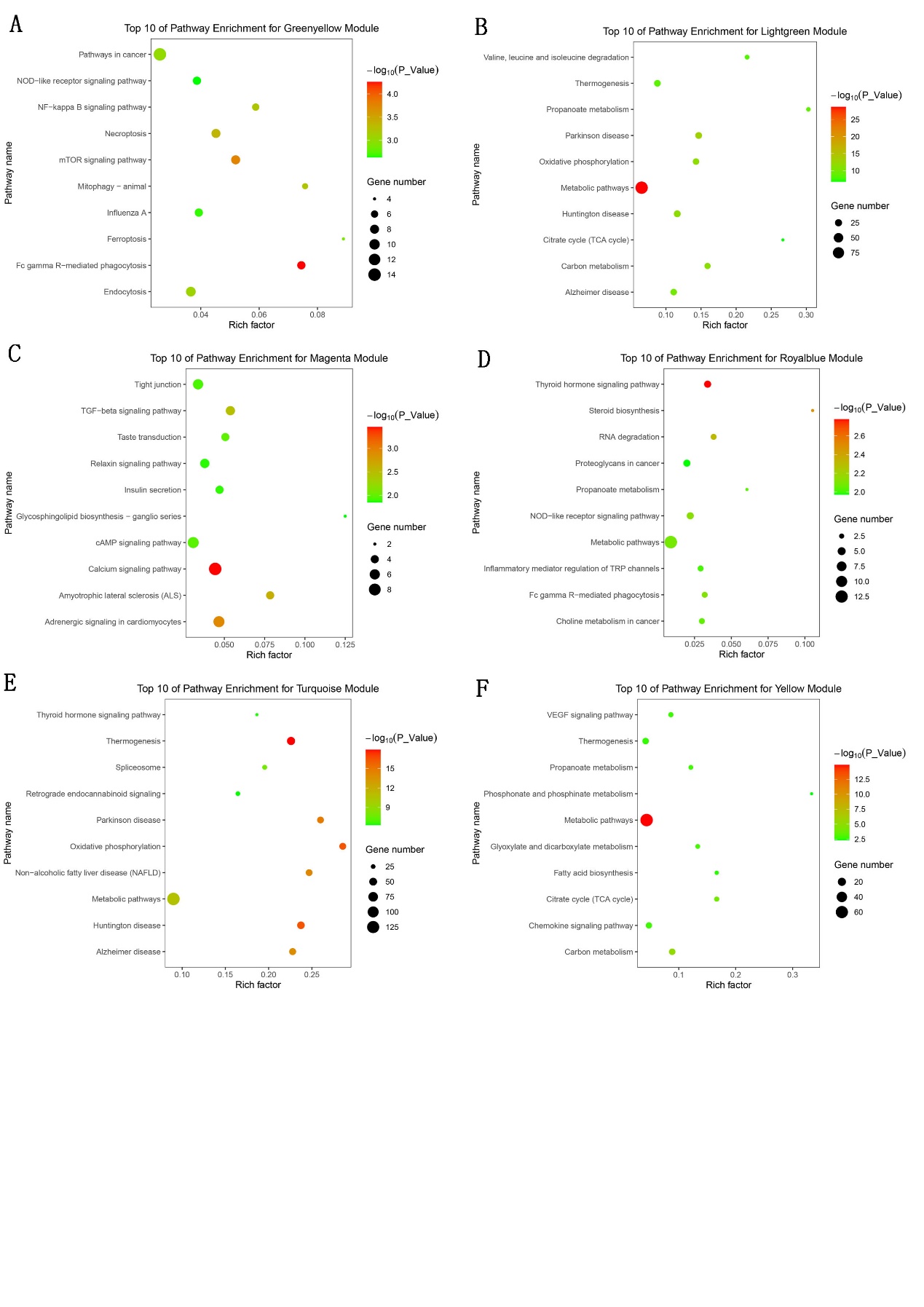
**

**Supplementary Figure 8.** KEGG pathway analysis of related DEGs. (A) The top 10 of pathway enrichment for greenyellow module. (B) The top 10 of pathway enrichment for lightgreen module. (C) The top 10 of pathway enrichment for magenta module. (D) The top 10 of pathway enrichment for royalblue module. (E) The top 10 of pathway enrichment for turquoise module. (F) The top 10 of pathway enrichment for yellow module.


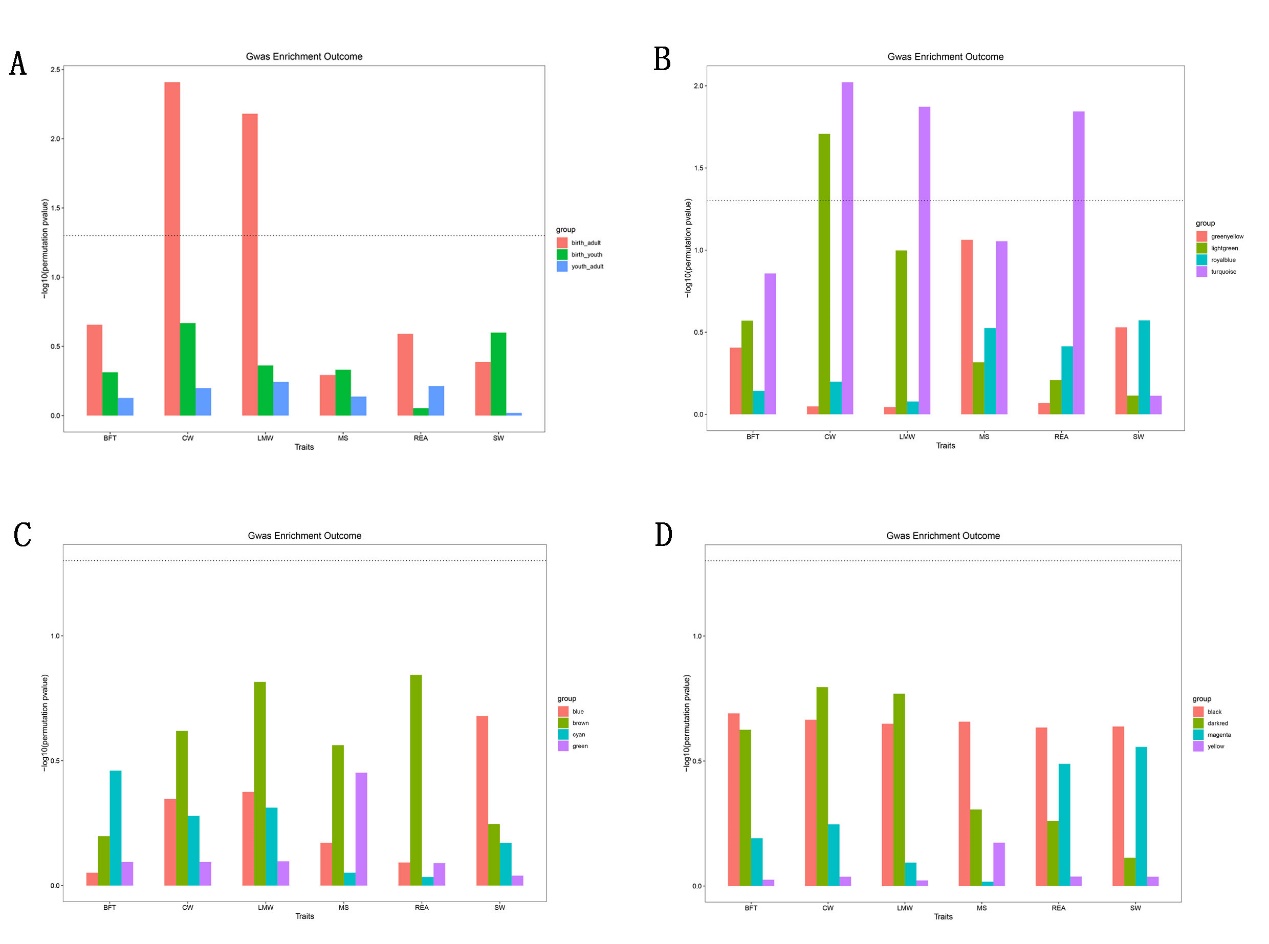


**Supplementary Figure 9.** The GWAS enrichment for DEGs in different groups. (A) The enrichment for DEGs of three comparisons. (B) The enrichment for DEGs of four modules including greenyellow, lightgreen, royalblue and turquoise. (C) The enrichment for DEGs of four modules including blue, brown, cyan and green. (D) The enrichment for DEGs of four modules including black, darkred, magenta and yellow. The line means P is equal to 0.05.
